# Abstracts of papers presented at the 2002 Pittsburgh Conference

**DOI:** 10.1155/S1463924602000184

**Published:** 2002


Journal of Automated Methods & Management in Chemistry
Vol. 24, No. 5 (September–October 2002) pp. 131–170

Taylor & Francis

Taylor & Francis Group

### Abstracts of papers presented at the 2002 Pittsburgh Conference

The following 131 abstracts form Part A of two issues of *Journal of Automated Methods & Management in Chemistry* devoted to abstracts of papers and posters presented at the Pittsburgh Conference held from 17 to 22 March 2002 in New Orleans, LA, USA. The papers and posters covered a range of topics and techniques, each of which provided valuable information to the conferees and exhibitors alike. The attendance figures remained disappointing, but Pittcon is still one of my favourite shows and has done a great job for Analytical Chemistry as a whole. People are tiring of New Orleans as a venue and it has become little more than a trade show. Hopefully, it will revive itself when the next show is held in Orlando, FL, from 9 to 14 March 2003. Perhaps the attendees will spare some time from Disney and Sea World to visit us at the exhibition and conference.

If you need further information about any of these abstracts, please contact the authors. It is my intention to publish full copies of some of these abstracts in future issues of the journal.

### Integration of the laboratory into the corporate knowledge bank

*Michael H. Elliott, Scientific Software, Inc., 6612 Owens Drive, Pleasanton, CA 94588-3334, USA*

Today's fast moving corporate environment requires business decisions to be made at an ever increasing rate. To make such decisions, such as whether to move forward with a product in development or to release a product to a customer, professionals need access to information scattered across the corporate enterprise. Laboratory information is critical to these business decisions in companies such as pharmaceutical and chemical manufacturers. Commercially available Laboratory Information Management Systems (LIMS) have been available since the early 1980s. LIMS have been very effective in increasing the productivity and sample throughput of the modern laboratory. However, they are only one piece of the puzzle to building an effective bridge between the laboratory and the rest of the corporate enterprise. Additional information, such as that from statistical packages, MSDS, COA, presentations, supply chain management systems, spreadsheets or other sources of laboratory knowledge need to be integrated within the business enterprise. This knowledge bank protects the intellectual property of the corporation as well as enabling fact-based decision-making. To enable this corporate knowledge bank, an integrated laboratory Knowledge Management System (KMS) must be in place to aggregate and integrate information from all these different sources. This electronic library must also be easily accessible to all those who need access to these knowledge assets for making fact-based decisions, insuring intellectual property, streamlining work flows or integrating disparate geographic locations. This knowledge bank must also be able to store non-laboratory data and easily integrate with other enterprise-wide systems, such as ERP. For the regulated environment, the consolidation of this enterprise information must comply with regulatory requirements such as US 21 CFR Part 11. This paper will discuss and demonstrate the advantages of integrating all laboratory information into the corporate knowledge bank. Examples of integrating all laboratory information and data and the subsequent integration of these assets into the corporate knowledge bank will be presented. Searching, accessing and viewing of this integrated information as well as how organizations can improve compliance with 21 CFR Part 11 will also be discussed.

### Using information technology to optimize product development in process industries

*Andrew Cunningham, Formation Systems, Inc., 144 Turnpike Road, 310 Southborough, MA 01772-2121, USA*

The best way to optimize your Laboratory Information Management Systems (LIMS) productivity may be not to test! See how product lifecycle management can reduce redundant testing and allow testing groups to focus on the highest ROI opportunities. To increase product performance, move from unique formulations to product platforms or just increase testing throughput. Companies are looking to automate sample creation using robots and move toward commentarial testing techniques. Both investments will increase the volumes of tests processed. These investments are based on assumptions that all test requests are required and these tests are not duplicate efforts. When formulation, material and test history are not readily accessible, formulators assume the shortest path is recreating the wheel. This leads to buying new raw materials when equivalent materials have been certified or when creating duplicate formulas. The real impacts are felt in downstream intesting where redundant testing is performed that results in wasted costs, lost testing productivity and longer development lead-times. Too often R&D departments have conflicting priorities and standalone systems that lead to gross inefficiencies, higher costs and lost orders. To optimize your LIMS investment, effectively managing the product requirements process and reuse of existing formulas and test results can reduce the volume of test requests and often eliminate whole testing cycles. Testing can focus on high-return opportunities rather than simply executing more tests. Formation Systems is the leading provider of Product Lifecycle Management (PLM) solutions for the CPG and process industries. Our Optiva® Software Suite automates product development and commercialization, reducing the time and resources required to take a product from idea through product launch. The results are accelerated revenue, lower costs, a simplified supply chain and a product development organization that enhances your ability to compete. Optiva includes powerful formulation, regulatory, specification and labelling functionality that complements a LIMS system. Formation's CPG customers use Optiva in addition to commercial or in-house LIMS systems. Insure LIMS testing is focused on highest ROI PLM solutions include both commercial and technical scoring technique to prioritize highest ROI projects and kill low probability requests. By prioritizing highest ROI opportunities and insuring redundant testing is largely eliminated, testing capacity is effectively increased.

### Rapid screening of environmental test wells by headspace mass spectrometry

*Brian G. Rohrback[1] and Elizabeth A. Harvey[2], [1]Infometrix, Inc., PO Box 1528, Woodinville, WA 98072-1528, USA, [2]Chevron Research, 100 Chevron Way, Richmond, CA 94801-2016, USA*

Gas chromatography is the preferred method for characterizing and quantitating low molecular weight compounds, but the separation process is often slow and often requires sample pretreatment. By eliminating the chromatograph and applying multivariate techniques to the bulk detector signal, composition can be determined quickly. Using a headspace sampling system with direct injection into a mass spectrometer, both qualitative and quantitative precision are demonstrated for a variety of environmental test applications. The short (3–5 min) analysis time and the lack of sample preparation creates a fast screening technique for fugitive hydrocarbons. Although headspace technologies have gained some acceptance in the flavour and fragrance industry as a means of evaluating product and ingredient quality, little has been done to assess feedstocks, products or wastes in the petroleum industry. Data for this study are drawn from a set of more than 200 test wells positioned at various locations within the boundaries of a refinery. For traditional quality assessment, classification techniques (such as nearest-neighbour and factor-based groupings) can be used to determine the contaminant origin even in cases where the candidate sources are chemically similar. Without resorting to GC separation, the concentration of classes of contaminants can be estimated over a four order-of-magnitude range.

### Automation of field-deployed sensors for long-term monitoring

*Scott R. Burge, Burge Environmental, Inc., 6100 S. Maple Avenue, 114, Tempe, AZ 85283-2872, USA*

The research was performed to ascertain if a variety of sensors including ion-specific electrodes, photo-ionization and a trichloroethene (TCE)-specific optrode could be deployed in the field to perform long-term monitoring of contaminated ground-water systems. A 'universal' sensor platform was designed and fabricated which allowed the deployment of the sensors. The platform allows the sensors to be installed in the water sample or in the headspace above the water sample. The platform could be interfaced to accommodate a variety of ground-water pumping systems and was designed to derive its power from a solar cell. The platform could be installed adjacent to or inside the monitoring well. The operation of the platform includes sampling, purging and cleaning of the system, calibrating the analytical systems and quality control checks such as duplicates and spiked duplicates. The platform is an automated analytical instrument, with protocols similar to those designed by the USEPA (i.e. SW-846), capable of performing analyses in the field with no operator. The system was field-tested at Homestead Air Force Base, Florida. The system was configured to allow the TCE optrode to measure concentrations in the headspace. The system sampled ground water from a shallow aquifer and analysed for TCE at a concentration of 70–100 ppb in the presence of several other volatile chlorinated compounds. No interference was observed from the other chlorinated compounds with TCE. The results of the system were comparable with the results of samples submitted for laboratory analysis (EPA Method 8260B.

### Rapid screening for VOCs in soil using a portable membrane inlet mass spectrometer (MIMS)

*Yoshihiko Arita[1], Takashi Hirano[1], Masahiko Fujiwara[1], Daisuke Satake[1], Koichiro Matsuda[1] and Steve J. Mullock[2], [1]Horiba Ltd, 2 Miyanohigashi Kisshoin Min, Kyoto 601-8510, Japan, [2]Kore Technology Ltd, 291 Cambridge Science Park, Cambridge CB4 0WF, UK*

Soil pollution from industrial development has become a source of concern more recently, starting in the 1980s when toxins in soil began increasingly to be detected at industrial sites. In Japan, the significance of the role soil plays in the natural environment has finally been recognized and the public is rapidly becoming more aware of the contaminated soil problem. In the soil analysis industry, both portability and sensitivity at high speed are essential for the success of preliminary site survey work. The traditional methods for site screening such as detector tubes and portable GC-PIDs are not sufficiently sensitive to detect ppb levels in soil. In addition, as many measurements are generally required to characterize a site, a more rapid analysis is highly advantageous for the preliminary survey. The MS-200, portable time-of-flight mass spectrometer with membrane inlet designed by Horiba and Kore Technology allows much more rapid analysis in $< 5$ min, with higher sensitivity than GC, for VOCs in soil and semi-real-time analysis in ambient air. The MS-200 was evaluated for a number of different soil samples. In particular, rapid analysis, using mixture analysis to perform a least-squares fit of library data for complex mixtures instead of separation by gas chromatography, will be discussed. The most striking feature of the MS-200 is undoubtedly the speed, but its sensitivity at ppb levels and accuracy should also be noted and will be discussed. The results of these experiments will be presented, backed up by data from a GC. In this paper, we will discuss the future potential, as well as the current performance, of MIMS.

### Monitoring atmospheric particulate matter through cavity ringdown turbidimetry

*Jonathan E. Thompson, James D. Winefordner and Benjamin W. Smith, University of Florida, Department of Chemistry, PO Box 117200, Gainesville, FL 32611-7200, USA*

The study of micrometer- and nanometer-sized suspended airborne particulate matter has become increasingly important in recent years. The interest in the aerosol has been driven by the revelation that airborne particulate matter adversely affects human health, as well as its possible role in the global energy balance. In this work, we have developed an instrument based on the cavity ringdown technique capable of monitoring atmospheric extinction in real-time. As the majority of atmospheric extinction is often caused by aerosol scattering/absorption the technique is useful for monitoring suspended particulate matter. We have applied this technique to monitoring changes in extinction following smoke from a nearby wildfire and have also correlated extinction measurements with levels of particulate mass at our location.

### Identification of citrus flavonoids by liquid chromatography: time-of-flight mass spectrometry using accurate mass measurement and nozzle-skimmer fragmentation patterns

*Rick Parry[1], Evaldo de Armas[2] and Jack Cochran[3], [1]LECO Corporation, Separation Science Applications, 3000 Lakeview Avenue, and [2]Marketing, 4000 Lakeview Avenue, Saint Joseph, MI 49085-2319, USA, [3]LECO Corporation, Separation Science, 815 Pilot Road, Ste C, Las Vegas, NV 89119-3739, USA*

Flavonoids found in fruits and vegetables have been shown to provide a variety of medicinal benefits in the areas of cancer treatment and prevention as well as the reduction of allergic response. The analysis of flavonoids is complicated by the complexity of the food matrices in which they are monitored. The rather low concentrations of these analytes are easily masked by other matrix components significantly complicating the accurate identification of the flavonoids. A variety of chromatographic techniques have been used to attempt to address this analytical problem. More recently, LC-MS and LC-MS-MS approaches have been used to minimize further matrix interferences. However, other LC-MS tools such as accurate mass measurement, nozzle-skimmer fragmentation, and a combination of peak location and mass spectral deconvolution algorithms may provide significant advantages in the detection and proper identification of the flavonoids in complex matrices. In this paper, a group of seven flavonoids has been successfully identified in a citrus matrix using a commercially available LC-TOFMS system. Unique peak location and mass spectral deconvolution algorithms were used to simplify significantly the process of identifying the chromatographic positions of all analytes in the matrix and obtaining a pure spectrum for each analyte. Accurate mass measurement was used to determine potential empirical formulas for each analyte. Simple spectral interpretation techniques were also applied to determine reasonable elemental ranges for the formula calculator. Potential formulas were generated and then searched against a variety of commercial databases to obtain possible structures for the analytes. Finally, nozzle-skimmer fragmentation was used to minimize the number of potential identifications.

### Rapid, solvent-free fat analysis for food and dairy applications

*Vince Collins and Bobbie J. Haire, CEM Corporation, Applications, 3100 Smith Farm Road, Matthews, NC 28104-5044, USA*

Industries producing food or dairy products typically test for fat content. Many of these companies rely on the traditional methods such as Babcock, Mojonnier or Soxhlet extractions. These methods are long and laborious and require the use of various solvents. These procedures are not beneficial for process control needs. The SMART Trac System uses a new technology to provide rapid fat analysis for the food and dairy industries. This technology combines the use of microwave drying with nuclear magnetic resonance (NMR) to provide an accurate moisture and fat result in < 3 min for most samples. Prior work performed with NMR technology was subject to oversaturation of the signal with high moisture products. The use of microwave drying to remove moisture combined with NMR for measurement of fat protons allows accurate moisture and fat data. This test can provide valuable and timely information for the plants to use for process control. This paper discusses results comparing traditional methods and the new microwave/NMR technology for ice cream mixes, cream and dried dairy powders.

### Automated enzymatic food analysis

*Wilfried Liekmeier and Paul-Heinz Sollboehmer, Ascanis OHG, Theodor-Lachmannstr 17, Ueberlingen D-88662, Germany*

Enzymatic food analysis methods are highly specific and efficient and are indispensable for food analysis according to national and international norms and recommendations (e.g. IFU, EN, IDF and ASBC). The technique allows one to quantify important food constituents like sugars, fruit acids, cholesterol and others with minimal sample preparation. The practical implementation however sometimes seems tedious and complex. The technique is illustrated and it is shown how a new application software package substantially facilitates enzymatic analysis with commercial test kits and how it improves its results. The EnzLab software guides the user through the analysis, controls the measuring wavelength and times, it automatically reads the spectrometer data and performs all required calculations. Predefined analysis methods, configurable sample documentation and operators directions as well as a comprehensive analytical report are further items of rationalization of the technique. The improved timing accuracy will improve the result quality. A complete automated system is for example based on a standard double-beam UV/Vis-spectrophotometer—optionally with a 13-cell automatic cell changer for parallel sample measurements. The system can further be expanded with a piston pump for precise enzyme injection. High photometric accuracy of the system will decrease detection limits and reduce sample preparation requirements. For existing photometers without PC-communication, the 'manual' software version is applied. The software prompts the user according to the selected method/analyte and samples to enter the photometric readings manually via keyboard or clipboard.

### Smart screening of pesticides using double pro injection on the electron-capture detector and ion-trap GC-MS/MS

*Jessie Crockett Butler*[1], *Meredith Conoley*[1], *Don Clay*[1], *John Ragsdale*[1] *and Jim Edwards*[2], [1]*ThermoFinnigan Corporation, 2215 Grand Avenue Parkway, Austin, TX 78728-3812, USA,* [2]*ThermoFinnigan Corporation, 100 Busch Drive NE Cartersville, GA 30121-7217, USA*

The analysis of chlorinated pesticides is routinely performed on an electron capture detector (ECD) with a confirmational analysis by GC-MS. Since the ECD is a semi-selective detector for chlorinated pesticides, sometimes a false-positive will occur. Subsequent analysis by GC-MS is necessary to confirm the presence of the pesticide. The two analyses are usually done on two different GCs and by different analysts. Considerable time is spent in reviewing the data to determine which samples will require the confirmation by GC-MS. A software program has been developed to program the autosampler to inject the sample on the ECD for screening and then on the GC-MS for confirmation, only if a target compound is found. Intelligent software makes smart screening possible by combining an ECD and Mass Spec analysis on one GC. The Trace GC may be configured with an ECD, PolarisQ Ion Trap Mass Spec, dual split/splitless injectors and a fully programmable AS2000 autosampler. The injection of the sample on the ECD is made first to screen for the presence of pesticides. Then at the end of the run the data are processed. If a target compound is detected, the autosampler will re-inject the same sample on the GC-MS. A duplicate column is installed in the second injector to the PolarisQ Ion Trap. The mass spec is set up for analysis by MS/MS. In this mode of operation, the matrix interferences are excluded from the trap and a precursor ion is isolated. Then energy is applied to fragment the ion to form a complete spectrum of unique product ions for confirmation. This fast screening double injection technique has previously been used for analysis with dual ECDs where a screening method and analytical method or combined in one GC [1].

1. Clay, D., Automatic screening and analysis of samples using a single autosampler in double injection mode. ThermoFinnigan Corporation. Pittsburgh Conference, 2001 (2002).

### Simultaneous gas chromatography/mass spectrometry and gas chromatography/flame ionization detector

*James D. Hudson*[1], *Michael T. Cheng*[1] *and Carla Remigi*[2], [1]*Chevron Research and Technology Company, Petroleum Chemistry, 100 Chevron Way, 51-1142, Richmond, CA 94801-2016, USA,* [2]*Chevron Research and Technology Company, 445 Oxford Street, San Francisco, CA 94134-1619, USA*

Interest in having both Gas Chromatography/Mass Spectrometry (GC/MS) and Gas Chromatography/Flame Ionization Detection (GC/FID) data has created the demand to perform both types of analyses simultaneously using a single injection. This set-up gives the best of both worlds: GC/FID, which is deemed superior for quantitation, and GC/MS, which is advantageous for confirmation of compound identification based on the mass spectrum. A procedure to use dual columns from a single injector port of an HP 6890 GC coupled to two different detectors is described. The detectors are a FID and an HP 5973 mass spectrometer. The GC was interfaced to both the mass spectrometer and the FID. Two $100\,m \times 25\,mm$ i.d. $\times 5\,\mu m$ film thickness HP-1 (crosslinked methyl silicone gum) columns were connected to the rear injector port using a two-hole ferrule (Agilent Part # 5062-3580). The detector ends of the columns were connected in the usual manner. No other modification was necessary. The Chemstation software for the HP 5973 mass spectrometer is capable of handling the acquisition of both the FID and mass spectral data provided that the computer is of sufficient speed. For the analysis described below we used a $1\,\mu l$ injection and 100:1 split ratio. Since the sample is split between two columns, we injected approximately twice the amount desired per column. GC/MS and GC/FID traces of a gasoline sample, for the peaks elute earlier in the GC/MS trace. The difference in retention times is attributable to different column lengths that might have occurred upon manufacture or cutting during installation. Despite this difference, peaks are easily matched by comparing the pattern of peaks; therefore, we did not try to adjust column lengths to make these retention times coincide. It is possible that the vacuum of the mass spectrometer may draw more sample toward it than the FID, but this does not pose a problem because of the greater sensitivity of the FID.

### Improving gas chromatography sensitivity with multiple injections

*Alex Niewland*[1], *Steve L. Morgan*[1], *William E. Brewer*[2] *and Nathaniel T. Greene*[3], [1]*University of South Carolina, Chemistry and Biochemistry, 631 Sumter Street, Columbia, SC 29208-0001, USA,* [2]*Clemson Veterinary Diagnostic Center, Toxicology, 500 Clemson Road, Columbia, SC 29229-4306, USA,* [3]*University of South Carolina, PO Box 102406, Columbia, SC 29224-2406, USA*

Over the last years, the injection of larger-than-normal volumes in capillary gas chromatography (GC) has attracted an increasing amount of research attention. The objective of large volume injection (LVI) is the achievement of an increased signal-to-noise ratio by the introduction of more sample onto the column. This is particularly important in detecting analyte in trace amounts, such as in toxicological applications. However, LVI is still not common practice in most toxicology laboratories. Many large-volume injectors are commercially available. These injectors are designed to inject over 100 ml of sample volume, as compared with the conventional 1–3 ml. The injection port is generally set below the boiling point of the solvent, while the split vent is opened to divert the solvent flow. Subsequently, the injector temperature is rapidly ramped to a high temperature, with the split vent closed, and the analytes are swept onto the GC column. Some work has been reported on improving GC sensitivity by injecting several 100 ml of sample with a conventional split/splitless injector. A packed injection liner, with either silanized glass wool or Tenax TA, was used for the analysis. Also, the septum purge flow was set high throughout the injection and the initial oven temperature was set at least 20–30°C above the standard solvent boiling point to facilitate removal of the solvent. This method worked well with the various hydrocarbons tested. However, large losses of volatile sample components could occur [1]. Another viable method is to inject 1–2 ml of sample multiple times using a conventional split/splitless injector. For example, a splitless injection of 1 ml of sample can be repeated every 20 s for a total of 10 ml of sample volume, thereby improving GC sensitivity by a factor of five to 10 times. This improvement in sensitivity is often sufficient for many applied environmental and toxicological applications, especially with sample volumes of only 25–50 ml. Examples of this injection method are demonstrated for pesticides and drugs.

1. Grob, K. and Brem, S., *Journal of High Resolution Chromatography*, 15 (1992), 715.

### On-line electrolytic eluent generators and their applications in ion chromatography

*Yan Liu[1], Chris Pohl[1], Victor Barreto[2], Zhongqing Lu[3] and Nebojsa Avdalovic[2], [1]Dionex Corporation, Chemistry, 1228 Titan Way, Sunnyvale, CA 94085-4074, USA, [2]445 Lakeside Drive, Sunnyvale, CA 94085-4704, USA, [3]Dionex Corporation, Chemistry & Biotechnology Ctr Department, Box U-60, Storrs, CT 06269-3060, USA*

Dilute solutions of acids, bases and salts are commonly used as chromatographic eluents in ion chromatography. Traditionally, these eluents are prepared off-line by dilution from reagent-grade chemicals. The off-line preparation of chromatographic eluents can be tedious and prone to operator errors, and it often introduces contaminants that can cause undesirable chromatographic baseline artefacts and even irreproducible retention times of target analytes during the ion chromatographic separation. Therefore, there is a need for improved methods of preparing high-purity chromatographic eluents for ion chromatography. We have recently developed novel electrolytic devices that can be used to produce electrochemically high-purity electrolyte solutions for use as ion chromatographic eluents using de-ionized water as the carrier stream. In this presentation, we will describe the principles and operation of the automated on-line electrolytic eluent generators and demonstrate the advantages of using these eluent generators in isocratic and gradient ion chromatographic separations.

### *In situ* Raman investigation of 4-nitroazobenzene modified glassy carbon electrodes

*Takashi Itoh and Richard L. McCreery, Ohio State University, Department of Chemistry, 100 W. 18th Avenue, Columbus, OH 43210-1106, USA*

When coupled with electrochemical techniques, *In Situ* Raman spectroscopy is powerful tool for investigating electron-transfer processes in electrochemical reactions. This combination has proven very advantages in studying reactions occurring at the interface between electrode and electrolyte solutions. In this study, *In Situ* Raman investigations at potentiostatic condition were applied to a monolayer of 4-nitroazobenzene (NAB) molecules on glassy carbon (GC) electrodes (NAB-GC) in the acetonitrile (ACN) solution of $1_M$ tetrabutylammonium tetrafluoroborate (TBA-BF4). A special cell was designed and constructed for the simultaneous use of *In Situ* Raman and electrochemical measurements. The NAB-modified electrode preparation procedure in this work was similar to that described in the literature. The variation of NAB-GC Raman spectra in $1_M$ TBA-BF4 and ACN at the electrode potential (a) +400 mV, (b) −1200 mV and (c) −1500 mV (versus $Ag/Ag^+$) shows no spectral changes in the potential range between +400 and −800 mV. However, at a more negative potential range between −800 and −1800 mV, we could see spectral changes in the frequency range between 1100 and 1600 $cm^{-1}$. These spectral changes are attributed to reduction of the NAB monolayer to a chemisorbed NAB anion. We shall discuss such changes of *in situ* Raman spectra.

### Advances in mass-directed automated high-throughput sample processing

*Christopher P. Loran[1], William C. Schnute[1] and James A. Schibler[2], [1]Dionex Corporation, Mass Spectrometry Marketing, 500 Mercury Drive, Sunnyvale, CA 94085-4018, USA, [2]Dionex Corporation, 1228 Titan Way, Sunnyvale, CA 94085-4074, USA*

High-throughput screening (HTS) methodologies greatly reduce the time and costs associated with drug and chemical development. The many synthesis products generated from combinatorial chemistry techniques require purification and analysis before screening of their bioavailability, pharmacokinetics, toxicity and other biological activities. A fully automated high-throughput system for analytical and semi-preparative HPLC has been developed to incorporate mass-directed fraction collection for the rapid identification and collection of important fractions. New developments include fraction collector triggering based on any combination of detection parameters (including time, threshold, peak slope, and sensitivity) to ensure complete collection of the analytes of interest; and sample-specific control variables (e.g. mass) used to make real-time changes to control parameters without changing instrument control programs. The chromatography management system provides a single platform with consolidated control panels to control all the components of the HTS chromatographic system, track samples, collect data, and compare results from multiple screenings, with integrated system checks in a 21 CFR 11-compliant environment. In this presentation, we will show how recent advancements in instrumentation and software were applied to HTS methodologies. Examples of high-throughput LC/MS, and fast method development will be presented using ultra-fast gradients with mass-directed fraction collection from 96- or 384-well microtitre or deep well plates.

**Creating and using searchable mass spectral libraries with LC/MSD**

*A. Paul Zavitsanos and James D. McCurry, Agilent Technologies, 2850 Centerville Road, Wilmington, DE 19808-1610, USA*

The formation of various product ions observed in a mass spectrometer by collision induced dissociation (CID) is a function of variety of factors: energy (voltage and target gas molecular weight), collision frequency (pressure, cross-section of gas), time and the structure of the compound under study. This presentation will focus on technologies that allow the creation and use of meaningful API spectral libraries with the Agilent LC/MSD. A novel tune procedure is described and search results for a variety of compounds of environmental interest are demonstrated. The unique characteristics of the Agilent LC/MSD that make it such an ideal platform for this approach are discussed.

**A system for automated method development using liquid chromatographic mass spectral detection**

*Eduard Kolovanov[1], Mike McBrien[1] and Anthony Williams[2], [1]Advanced Chemistry Development, 90 Adelaide Street W., Toronto, Ontario, Canada M5H 3V9, [2]Advanced Chemistry Development, 225 Jamestown Road, Pittsboro, NC 27312-6752, Canada*

The principles behind computer-assisted chromatographic method development are well established. Experiments are performed in which one or more parameters are modified, and the changes in retention time for each peak are used to predict the best conditions under which to work. Predicted experiments are attempted, and the model is refined as necessary. The result is generally a better method developed in a shorter time frame. However, for particularly challenging samples, the experiment/prediction cycle may involve a large number of iterations, and a concurrently large amount of time and effort for the chromatographer. Attempts have been made to automate fully this process, hampered by inability to track (particularly impurity) peaks. Advanced Chemistry Development has designed a solution to this problem, by using secondary detection techniques. ACD/AutoChrom uses both UV-Vis and MS detection to track peaks unequivocally, developing methods resolving even trace impurities. The software is designed to interface with any of a number of compatible LCMS instruments, and is fully documented to allow integration with custom-built software.

**A rapid high-performance liquid chromatography method for the simultaneous analysis of multiple sunscreens in cosmetic products**

*Gaspare A. Albanese[1], Peter A. Landa[1], Peter Kaufmann[1], Asira Ostrovskaya[2] and Paul Cerasuolo[2], [1]Estee Lauder, Inc., Research and Development, 125 Pinelawn Road, Melville, NY 11747-3145, USA, [2]Estee Lauder, Inc., Special Analytical Services, 125 Pinelawn Road, Melville, NY 11747-3135, USA*

It is well known that long-term repeated exposure to UV-A and UV-B radiation from the sun is extremely harmful. Long-term exposure is known to cause skin photo-ageing, sunburn, photosensitivity and an increased risk of skin cancer. As a result, many chemical and physical sunscreens have been incorporated into cosmetic formulations to combat the effects of UV-A (320–400 nm) and UV-B (290–320 nm) radiation. Therefore, the analysis of sunscreens in cosmetic formulations has become an important issue. Many recent publications have addressed the issue of sunscreen analysis. Most methods involve performing multiple chromatographic runs and the use complicated buffer systems to acquire the desired separation of the analytes of interest. We have developed and optimized an HPLC method for the separation and quantitation of several of the most commonly used sunscreens: benzoresorcinol, 2-hydroxy-4-methoxy benzophenone, oxybenzone, avobenzone, octyl methoxycinnamate, octocrylene and octyl salicylate. Our new approach reduces the actual number of chromatographic analyses needed into a single 25-min run. The method uses a non-buffered gradient system with a reverse phase C8 column. Use of a scanning photo-diode array detector enables each component to be extracted and quantified at its respective wavelength maximum. Accurate quantitation is accomplished using an external standard and identification is confirmed by UV spectral analysis. The use of this newly developed method has dramatically increased productivity and efficiency in the laboratory.

**Real-time product and process control in pharmaceutical manufacturing by using the Brimrose acousto optic tuneable filter near infrared technology**

*Gabriel Levin[1], Igor Nazarov[1] and John L. Hill[2], [1]Brimrose Corp. of America, Spectrometer Division, 5024 Campbell Blvd, Baltimore, MD 21236-5974, USA, [2]Brimrose Corp. of America, 119 Radcliff Cir, Durham, NC 27713-2504, USA*

The issue of drug quality and security has been the focal point for endless efforts ever since drugs were manufactured. NIR has been implemented in several ways from incoming materials inspection to blend uniformity control. Implementation was hampered by low speed, sensitivity to ambient light variations, vibrations. Over the past several years, Brimrose cooperated with major companies in providing successful on line solutions. The following are some examples.

- Drying in a fluidized bed is a complex process, real-time control is essential for reproducible and quality product. The result is reproducible product and consistent tableting.

- Vial contents and moisture are measured as they move on a belt, without stopping, or aligning the vials. Combination with colour code and barcode readers possible. Product is safer.
- Tablets are measured as they move on a belt, without stopping. Placebo versus active tablets are validated. Rates can vary from 10 to 60 per min. Instruments are available with simultaneous reflectance and transmission capability.
- Quality and uniformity of transdermal patches is critical. Real-time inspection is implemented on moving patches. The results are huge savings and excellent quality.
- Tablet coating is crucial in many instances, measurement is made on line contents.
- Blending uniformity is determined with the Brimrose battery operated analyser that rotates with the blender. Uniformity algorithms are implemented in real-time. Process development cycle and cost is dramatically reduced, along with safer products.

### Applications of a portable FTIR analyser system in the pharmaceutical industry

*John P. Coates, Coates Consulting, 12 N. Branch Road, Newtown, CT 06470-1858, USA*

Infrared spectroscopy has an important and unique role in the analysis and quality control of pharmaceutical products. Its ability to provide a unique fingerprint of a material makes it ideal for the definitive identification and characterization of drug materials and associated raw materials. The technique can be, or has the potential to be, applied successfully to all facets of the industry, from raw material quality control to field characterization of formulated final products/dosage forms. The role of infrared spectroscopy has traditionally been limited because of the industry requirement for absolute reproducibility of measurements, a factor that has been an issue with traditional infrared measurement techniques. The use of diamond ATR simplifies sample preparation makes it practical to sample materials equally in all forms: from liquid reactants to solid excipients, and from aqueous-based injectables to tablet and capsule formulations in final dosage forms. The diamond-based concept of sample preparation now provides a single method of sample handling that can be applied essentially to all materials without imposing changes in form or composition. Furthermore, the technique is very reproducible and requires no special skill in its operation. This paper will discuss the use of diamond ATR in an integrated, portable system for the following.

- The screening and testing of incoming raw materials, which can now take place at the point of delivery, the warehouse facility and/or the product blending area.
- The role as an R&D and production tool in various areas of the plant, including reaction vessels, fermentation vats and product dryers will be covered. In this latter case, it is possible to move the system on a cart to the specific point of the process, and provide fresh data, without the delays associated with sending samples back to a remote analytical laboratory.
- Finally, dosage forms can be tested for consistency, quality, and even identity from the warehouse, to the distribution centre, to the drug store or the hospital pharmacy. It will be demonstrated that this approach, with a combination sample handling and instrumentation, can provide a universal tool for the characterization of raw materials, intermediates and final products in the pharmaceutical industry.

### Particle-based solid-phase nano-extraction for protein and etabolic profiling using nano-barcode identification tags

*Lin He, Remy Cromer, Rajendra Singh, Christopher Becker and Michael J. Natan, SurroMed, Inc., Chemistry, 2375 Garcia Avenue, Mountain View, CA 94043-1104, USA*

The development of techniques and methods for efficiently separating and detecting analytes in biological fluids becomes increasingly important in the post-genomic era. We have previously described a novel platform coupling self-encoded metal particles with mass spectrometry in the field of immunoassays, relying on high-affinity capture of analytes from biological matrices before MS analysis. Herein, we report an expanded application of these particles as a solid-phase extraction (SPE) medium in biological analysis. Nanobarcodes identification tags (NBCs) possessing varying surface chemistries are used to extract rapidly classes of metabolites/proteins from biological fluids in the presence of high levels of salts and abundant proteins. A large variety of stationary phases are tested, ranging from traditional chromatographic materials to biomolecules covering a wide range of affinities. Captured analytes, including small molecules, peptides and proteins, are detected using both MALDI and ESI mass spectrometry after elution and are identified by MS/MS. Thus, multiplexed comprehensive profiling of biological samples is carried out using NBCs derivatized with a combination of different stationary phases. In addition, an automated system is evaluated to improve the throughput of sample processing. The results presented will further illustrate the capability of this novel NBC-MS interface to achieve highly multiplexed, sensitive and selective measurements in small volumes of biological samples.

### A novel molecular aptamer beacon for real-time protein recognition

*Jinawei Jeffery Li*[1], *Weihong Tan*[1] *and Xiaohong Fang*[2], [1]*University of Florida, Department of Chemistry, PO Box 117200, Gainesville, FL 32611-7200, USA,* [1]*University of Florida, Institute of Chemistry, 52 Sanlihe D, Beijing 100080, PR China*

One of the most pressing problems facing those attempting to understand the regulation of gene expression and translation is the necessity to monitor protein production in a variety of metabolic states. Yet, there is no easy solution that will either identify or quantitate proteins in real-time. Recently molecular beacon-based assays have shown promise for real-time protein detection. Here we introduce a novel approach to construct an aptamer beacon for real-time protein recognition and quantitation. An aptamer beacon based on a thrombin-binding aptamer was prepared by labelling the two ends of the aptamer with a fluorophore and a quencher, respectively. The aptamer beacon combines the signal transduction mechanism of molecular beacons and the molecular recognition specificity of aptamers. Significant fluorescent signal change was observed when aptamer beacon was bound to thrombin, which is attributed to a significant conformational change in aptamer beacon from a loose random coil to a compact unimolecular quadruplex. The aptamer beacon recognizes its target protein with high specificity and high sensitivity in homogeneous solutions. Ratiometric imaging has been conducted with aptamer beacon labelled with two fluorophores, which makes it feasible for protein quantitation in living specimen. The unique properties of the aptamer beacon will enable the development of a class of protein probes for real-time protein tracing in living cells and for efficient biomedical diagnosis in homogeneous solutions.

### On-site assessment of contaminated sites: application of sensors for site characterization

*Katrin Batereau, Baldur Barczewski, Norbert Klaas and Martin Müller, University of Stuttgart, Pfaffenwaldring 61, Stuttgart D-70550, Germany*

The main objective of the research is the development of field-screening instruments for on-site and *in-situ* measurement and assessment of contaminated sites to increase the statistical certainty of site characterization (by renunciation of detail precision of single-point measurement) and decrease costs for investigation. The used sensor arrays consist of different sensor types of varying sensitivities. Data analyses by pattern detection give information about chemical composition and the concentration of contaminants. Metal oxide and quartz oscillators are suitable for measurement of VOC in the subsurface. The prototype that contains both sensor types is fitted to a geotechnical driving rod and show—in laboratory and field tests—sufficient sensitivity to detect of contamination typically found in the field. The first attempt of pattern detection showed that the distinction between chlorinated and mineral oil hydrocarbons is possible. Laboratory and field tests showed that fibre-optic sensors are suitable for the detection of free-phase organic contamination and of dissolved organic contaminants (e.g. PAH). Further tasks are investigation and application of different sensor principles (surface wave resonators, heat conductivity sensors, etc.).

### A real-time ratiometric method for the determination of glucose inside living cells using optical nanosensors

*Hao Xu[1], Raoul Kopelman[1] and Jonathan Aylott[2], [1]University of Michigan, Department of Chemistry, 930 N. University Avenue, Ann Arbor, MI 48109-1001, USA, [2]Department of Chemistry, University of Hull, Hull HU6 7RX, UK*

Polyacrylamide (PAA)-based, ratiometric, spherical, optical nanosensors, or PAA PEBBLEs (Probes Encapsulated by Biologically Localized Embedding), are made and demonstrated here to enable reliable, real-time measurements of intracellular glucose. The PAA nanosensors are prepared using a microemulsion polymerization process. The sizes of these spherical PEBBLE sensors range from about 20 to 200 nm. These sensors incorporate glucose oxidase, an oxygen sensitive fluorescent indicator $(Ru[dpp(SO_3Na)_2]_3)Cl_2$, and an oxygen insensitive fluorescent dye, Oregon Green 488-dextran or Texas Red-dextran, as a reference for the purpose of ratiometric intensity measurements. The enzymatic oxidation of glucose to gluconic acid results in the consumption of oxygen, which is measured by the oxygen-sensitive ruthenium dye. The small size and inert matrix of these sensors allow them to be inserted into living cells with minimal physical and chemical perturbations to their biological functions. Compared with using free dyes for intracellular measurements, the PEBBLE matrix protects the fluorescent dyes from interference by proteins in cells, enabling reliable *in vivo* chemical analysis. Conversely, the matrix also significantly reduces the toxicity of the indicator and reference dyes to the cells, so that a larger variety of dyes can be used in optimal fashion. Furthermore, the PEBBLE matrix enables a synergistic approach in which there is a steady-state with local depletion of oxygen due to enzymatic oxidation of glucose. This cannot be achieved at all by pushing free enzyme and dyes into a cell. The work presented here describes the production and characterization of glucose sensitive PEBBLEs. The sensor response is determined in terms of linear range, response time, sensor stability and reversibility. The methods for loading PEBBLEs into cells will be discussed, including gene gun delivery and liposomal delivery. Applications of these PEBBLEs inserted in mammalian cells for real-time intracellular glucose analysis will also be demonstrated.

### Autonomous environmental monitoring devices: can potentiometric sensors play a role?

*Dermot Diamond, School of Chemical Sciences, National Centre for Sensor R, Dublin 9, Ireland*

The merging of wireless communications with chemical sensors and biosensors opens the way to the realization of minaturized autonomous sensing devices. While there are many potential areas of application for such devices, environmental monitoring is particularly attractive. Key characteristics for these devices can be summarized as able to function independently in the field for at least 1 year and capable of providing reliable analytical information throughout this period via wireless communications. Despite the emphasis in wireless communications on broadband systems for high-quality video and audio data exchange (e.g. Bluetooth), there have been significant developments in low-power, low-bandwidth systems that are much more suited to sensor applications. It is now possible to obtain relatively small transceivers commercially that can run for many months or even years on button batteries. However, for the concept to be realized, robust analytical systems are needed. Most attention is currently focused on lab-on-a-chip devices as they offer routes to on-line calibration, low reagent consumption and assays based on well-known chemistries. While potentiometric sensors have some attractive characteristics such as very low power consumption, signal independent of size (can be made very compact), flexibility in shape/size and good selectivity, there are formidable obstacles to be overcome if these sensors are to play a significant role in the emerging area of autonomous environmental monitoring devices. Chief among these are device lifetime, calibration, limit-of-detection and reference electrode/liquid junction design. These issues and the overall concept of autonomous monitoring devices will be discussed in this presentation.

### Nanoparticle ion-selective optodes for real-time subcellular chemical imaging

*Raoul Kopelman[1], Murphy Brasuel[1], Edwin Park[1], James Sumner[1], Terry Miller[2] and Martin Philbert[3], [1]University of Michigan, Department of Chemistry, 930 N. University Avenue, Ann Arbor, MI 48109-1001, USA, [2]University of Michigan, Environmental Health Science, 109 Observatory Street, Ann Arbor, MI 48109-2029, USA, [3]University of Michigan, Department of Toxicology, 1420 Washington Hts, Ann Arbor, MI 48109-2009, USA*

Ultrasmall, ultrafast, highly sensitive and highly selective PEBBLE (Probe Encapsulated by Biologically Localized Embedding) optical sensors have been produced in three classes of biocompatible matrices: hydrophilic, hydrophobic and amphiphilic. In addition to the original pH and calcium probes, these include now ratiometric potassium, sodium, magnesium, zinc and chloride nanosensors. While the hydrophilic (polyacrylamide) robes are based on selective fluoro-ionophores (pH, Ca, Mg, Zn), the hydrophobic (polydecylmethacrylate liquid polymer) probes enable highly selective operation via ion correlation (ion exchange or co-extraction). The hydrophobic nanospheres are stabilized (in solution or cell) by appropriate dispersers, as are the amphiphilic (sol-gel) PEBBLE nanoparticles. In spite of their nano-dimensions, the ion-correlation-based sensors exhibit calibration curves identical to those derived from thermodynamic equilibrium theory. While individual 200-nm sensors can be used, an ensemble of many such sensors is more practical for most bio-applications (and obligatory for 20-nm sensors). Reversibility and photostability are excellent. The overall intracellular lifetime of these disposable nanosensors is significantly longer than the required application time (cell lifetime), while the (dry) storage time is 6–12 months, or longer. Cell delivery is straightforward (by gene gun or liposomes) and can be directed toward subcellular compartments. Most importantly, these sensors are minimally cell invasive, both physically and chemically, while immune to chemical perturbations from the cellular environment. Real-time applications to brain cancer cells demonstrate the utility of chemical analysis and imaging by these nano-explorers.

### Spectroscopic characterization of nanomaterials at the single-particle and single-molecule level

*Joel M. Harris, David C. Hanley, Michael P. Houlne and Karla McCain, University of Utah, Department of Chemistry, 315 S. 1400 E., Salt Lake City, UT 84112-0850, USA*

The transport, adsorption and binding of organic molecules within porous-silica particles is important technology for chemical separations, bioanalysis, combinatorial synthesis and development of fluorescently labelled materials. The performance of these nanomaterials depends on understanding their chemical structure and reactivity, which are dominated by interfacial processes due to their high specific surface area. In this talk, several optical spectroscopy tools are discussed that can probe the structure of sol-gel silica materials and chemical reactions that occur within them. To observe the infrared spectra of adsorbates and bound molecules on silica surfaces, thin sol-gel coatings to an internal-reflection element, which provides a high surface area substrate for *in situ* ATR-FTIR experiments. Adsorption and binding of molecules to sol-gel silica surfaces have been investigated using this approach. For observing molecular transport within sol-gel materials, time-dependent fluctuations in fluorescence from a small population of molecules can be observed and related to diffusional kinetics within the sol-gel film. To gain a more detailed understanding of molecular transport in these materials, fluorescence of individual molecules can be imaged, and the trajectories of their random walks within the film can be mapped and characterized. Finally, the development of high-throughput optical microscopies allows individual particles to be observed and characterized. Fluorescence microscopy is employed to report dye labelling of individual nanoparticles at the single-molecule level. Confocal Raman microscopy is used to investigate the chemical structure and reactivity of individual, optically trapped submicron particles.

### A fast, accurate, on-line dilution accessory for flame atomic absorption spectrometry

*Steve Morton, Brian Clarkstone and Paul Neal, Thermo Elemental, Solaar House, Mercers Row, Cambridge CB5 8BZ, UK*

Flame atomic absorption spectrometry (FAAS) is a well-established, versatile analytical technique that is used widely in many industries. Although sensitive and selective, it suffers from a limited dynamic range. To overcome this, many types of sample require pretreatment and dilution to bring them into the measurement range of the technique, adding to the cost and complexity of the analysis. There have been several attempts to design an automatic dilution system that will perform this step of the analysis automatically at the point at which the sample is measured. These fall into two classes: those that mimic manual dilution, by mixing accurately measured volumes of the sample and diluent solution before passing the resultant mixture into to the spectrometer for measurement, and those that operate on a controlled flow principle, in which a sample stream with accurately known flow rate is dynamically mixed with a similar diluent stream at the sample inlet of the spectrometer. This paper is concerned with the second, dynamic mixing, and technique. Methods of creating flowing sample streams with accurately known and reproducible flow rates will be reviewed, and the advantages of a newly developed type of pump will be illustrated. The considerations involved in designing a practical dilution device based on these principles will be discussed, and the performance of such a device will be quantified with reference to some typical FAAS analyses.

### Determination of arsenic in environmental and biological samples using an electrothermal cell with tungsten coil in a hydride generation-atomic absorption spectrometry-flow system

*Solange Cadore*[1], *Anderson Schwingel Ribeiro*[1] *and Marco-Auréli Zezzi Arruda*[2], [1]*Chemistry Institut—Unicamp, Analytical Chemistry, Campinas, Sao Paulo, Sao Paulo 13087-970, Brazil,* [2]*Chemistry Institut—Unicamp, Building 735-A, Aiken, SC 29808-0001, Brazil*

The determination of trace elements by using tungsten coil as electrothermal atomizer and vaporizer has been made and shows to be a feasible alternative for the graphite tubes. Tungsten filaments show some advantageous characteristics such as: use of high temperatures (about 3500 K), non-porous coil surface and no memory effects, electric suppliers are only 150 W (e.g. 15 V, 10 A), no formation of refractory carbide and low cost. The aim of the present work was the determination of arsenic in natural waters and biological samples by hydride generation-atomic absorption spectrometry coupled with a flow injection system. The developed system uses a new electrothermal cell that employs a tungsten coil for the arsenic atomization, after the arsine generation. The parameters involved in the HG-AAS and merging zones flow injection systems were optimized: acid concen-tration (hydrochloric acid, 1 mol/l), reduction reagent concentration (sodium borohydride, 0.7% w/v), sample and reagent volumes (1 ml), atomization temperature (1900 K), reaction coil (60 cm long $\times$ 0.8 mm i.d.), flow rate for reduction reagent, HCl and sample (4.5 ml/min) and flow rate for carrier gas (250 ml/min). To improve the sensitivity the water vapour must be removed with a continuous system using a Nafion membrane, that allows a 92.7% efficiency. The calibration graph is linear up to 320 ng/ml arsenic ($R = 0.9999$). The limit of detection, calculates as three times the standard deviation of the blank signal, was 1.9 ng/l with relative standard deviation < 6%. The analytical frequency of the proposed system was 60 determinations per hour. It was possible to use four different types of tungsten coil with similar performance related to sensitivity and limit of detection. The proposed system did not showed interference from other elements, considering the species usually present in environmental and biological samples. The accuracy was evaluated with reference biological and environmental samples and the achieved results were in good agreement with certified values. The recovery of arsenic in mineral water and seawater, using the proposed system, showed values from 99 to 103% [FAPESP, CNPq].

### Direct and rapid determination of heavy metals in human blood and plasma by Zeeman modulation polarization atomic absorption spectrometry with high-frequency modulation

*Evgenia M. Iakovleva*[1], *Natalia B. Ivanenko*[1], *Anatolij A. Ivanenko*[1], *Marina Sherbaeva*[1] *and Alexander A. Ganeev*[2], [1]*St-Petersburg State University, Chemistry, Universitetskij Pr., St Petersburg, Russia* 198904, [2]*St-Petersburg State University, Chemical Research Institute, Moskovskij Pr. 19, St Peter, St Petersburg, Russia* 198004

Information about concentrations of elements in biosamples can be useful for medicine if we know the background levels, the ranges of changing and trends of the elements concentrations for healthy people and patients. It is desirable to use direct and rapid technique for this data obtaining. New technique Zeeman Modulation Polarization Atomic Absorption Spectrometry with High-Frequency Modulation allow one to determine many elements directly (without additional compounds and reagents or with there minimum use) in blood, plasma. The highest selectivity of this technique allows one to determine the elements in the presence of substantial background absorption—up to 98%. These parameters of the device allow carrying out the analysis of biological tests without preliminary pretreatment. For biosamples analysis (if we use atomic absorption technique with electrothermal atomization) two usual problems must be decided: removing the matrix effects and compensating on of background absorption. The matrix effects can be reduced in great deal with the aid of L'vov platform, sample pyrolysis and palladium modifier using. A high level of background absorption can be corrected by high selective Zeeman technique only. NIST blood standards were used in our work. The contents of metals were determined in human blood and plasma. One of these elements was Ca, but Ca concentrations in blood and plasma are very high and the sensitivities for this element are also very high. It demands a high degree of dilution. For ions resonant lines the sensitivities are determined by atomization temperature since the sensitivities one can change in high degree—up to 100 times. In this case, one can use rapid and high-precision technique almost without dilution. Because we have using ionic absorption spectrometry for analysis Ca. The data for trends (Pb, Mn, Cu, Al and Ca) are also presented. Copper and Al trends are almost absent. Mn concentration decreases in 1.5–2.0 times from morning to night. For 60% of subjects, morning Pb concentration is 60–90 ppb. Night concentration is low: from 30 to 70 ppb (because of liquid exchange). Subject no. 5 was working with lead—for this case, Pb concentration was high (168 ppb). Some metals (e.g. Ni, Cu, Al) were determined in the urine of children from two St Petersburg areas. The same metals we determined in the urine of children living in a resort area of the city. These results are discussed in the report.

### The determination of ultra-trace mercury in environmental samples by cold vapour atomic absorption and mercury concentrator

*Christine M. Rivera*[1] *and Matt McCrum*[2], [1]*GBC Scientific Equipment, Inc., Applications, 3930 N. Ventura Drive, Ste*

*350, Arlington Heights, IL 60004-7494, USA, [2]GBC Scientific Equipment Pty Ltd, Marketing, 12 Monterey Road, Dandenong, Victoria, Australia 3175*

This talk will describe the determination of mercury at the subparts-per-billion level using the technique of concentrating mercury onto a gold trap to form a gold–mercury amalgam. A hydride generator will be used to form the atomic mercury vapour, which will then pass to a gold ribbon. The mercury is vaporized from the surface and passed to a quartz cell for atomic absorption measurement. By adding the amalgam stem into the system, the sensitivity can be increased by 50 times. The technique will be explained including hardware schematics, as will the analytical procedures required for ultra-pure determinations of mercury. Sediments, standing waters and soils will be analysed. Sample data, detection limit and sensitivity will be presented.

### Determination of Hg in environmental samples using a spectrometer with dual atomic fluorescence detectors to meet the demands of US EPA Methods 1631 and 245.7

*David L. Pfeil and Arthur Reed, Leeman Labs, Inc., 6 Wentworth Drive, Udson, NH 03051-4918, USA*

Efforts to understand and control mercury pollution have expanded investigations into relatively pristine environments, pushing detection limit requirements into the subpart-per-trillion range. The Environmental Protection Agency has promulgated purge and trap methodology (1631) capable of achieving such limits. Method 1631, as published, employs manual sample and reductant introduction, demanding significant analyst interaction. Furthermore, with the improved sensitivity of Method 1631 comes an increased risk of contamination by samples that have an unexpectedly high concentration of mercury. In this work, we evaluate the analytical capabilities of an automated cold vapour atomic fluorescence system with the ability selectively to use gold amalgamation to enhance detection limits. By using two independent fluorescence detectors, the analyser provides an unparalleled working range for mercury determinations (from 0.5 to 200 parts-per-trillion without gold amalgamation and from 0.05 to 25 parts-per-trillion with gold amalgamation). In addition, each sample can be screened using the traditional sensitivity detector (normally used for Method 245.7 determinations). Only if the fluorescent intensity is below a preset level, will the instrument then analyse the sample using the amalgamation units and the high sensitivity detector, thus preventing contamination of the critical amalgamation units and the high sensitivity fluorescence cell. The system is capable of routinely providing detection limits typically $\leq 0.05$ ppt for compliance with US EPA Method 1631 and $< 0.5$ ppt for compliance with US EPA Method 245.7.

### Pushing the limits of ultra-trace mercury determination with cold vapour atomic absorption spectrometry

*Doug Shrader[1], Cathleen Zimmerman[2] and Christine Sartoros[2], [1]Varian, Inc., Optical Spectroscopy Instruments, 201 Hansen Ct, Ste 108, Wood Dale, IL 60191-1151, USA, [2]CETAC Technologies, 14306 Industrial Road, Omaha, NE 68144-3334, USA*

For over 30 years, the Cold Vapor Atomic Absorption Spectrometry (CVAAS) technique has been used for the determination of low levels of mercury in environmental, biological/clinical, mining and industrial samples. It has been the method of choice for analysts around the world. Recently the cold vapour technique has been modified to include fluorescence detection. Despite pressure from these Cold Vapor Atomic Fluorescence Spectrometry (CVAFS) methods, which can achieve ultra-low detection limits, CVAAS continues to be an extremely sensitive and reliable technique for mercury determinations. A new enhanced CVAAS instrument and software module will be discussed. This dedicated atomic absorption-based mercury analyser demonstrates improved mercury detection limits and linear dynamic range. These improvements have been made possible by the optimization of spectrometer hardware components and experimental parameters. A detection limit of 0.2 ng/l is achieved without the use of gold amalgamation, which can lead to memory effects when higher-level samples are measured. Analytical results from a variety of environmental and biological reference materials will be presented showing the enhanced capability provided.

### Monitoring brain metabolism in critical care patients using an on-line electrochemical assay

*Mark C. Parkin[1], Sarah E. Hopwood[2], Anthony J. Strong[2], Stuart Hibbins[3] and Martyn G. Boutelle[1], [1]Department of Chemistry, The Strand, London WC2R 2LS, UK, [2]Institute of Psychiatry, Manresa Road, London SW3 6LX, UK, [3]Academic Department of Neurosurgery, King's College London, London SE5 9PJ, UK*

We have successfully monitored levels of glucose and lactate in the brains of head injury patients over periods of up to 5 days in the intensive care unit. During this prolonged period following the initial injury, profound secondary damage to the brain can occur. Levels of extracellular glucose and lactate could provide an important marker of the energy state of the at risk tissue. Measurement of rapid changes in these levels could give vital information about the dynamics of energy supply within the brain. In patients, the levels are sampled using clinical microdialysis with the probe placed in 'at risk' brain tissue. Joining the probe to the on-line assay is a length of low volume tubing giving a 9-min lag from patient to assay. To achieve the high time resolution required, we designed a rapid sampling flow injection analysis system (FIA) coupled to low volume enzyme microreactors and electrochemical detection [1]. At the heart of the system is a custom-built dispersion valve that alternately injects nanolitre segments of dialysate stream into the independent glucose and lactate assays. Each assay consists of immobilized substrate oxidase and horseradish peroxidase enzyme. The enzyme turnover is mediated by a ferrocene derivative and amperometric detection is accomplished by a downstream electrode. At a microdialysis flow rate of 2 l/min this gives a sampling interval of 6 s for each pair of glucose and lactate measurements. Basal dialysate glucose levels varied between 100 and 1300 $\mu$M with a typical daily variation of 500 $\mu$M.

Basal dialysate lactate levels varied between 300 and 2000 $\mu_M$ with a typical daily variation of 200 $\mu_M$. Superimposed on these global changes were rapid increases and decreases in levels with a sub minute time scale. We think these changes reflect the brain capacity to resist both physiological and pathological challenges.

1. Georganopoulou, D. G., Carley, R., Jones, D. A. and boutelle, M. G., 2000, *Faraday Discussions*, 116 (2000), 291.

### Luminescent zinc nanosensor for real-time monitoring of cellular processes

*James P. Sumner[1], Carol Fierke[1], Raoul Kopelman[1] and Jonathan Aylott[2], [1]University of Michigan, Department of Chemistry, 930 N. University Avenue, Ann Arbor, MI 48109-1001, USA, [2]University of Hull, Department of Chemistry, Hull, UK*

The development and characterization of a fluorescent PEBBLE nanosensor for the detection of cellular zinc is detailed. Zinc has been implicated in a number of events including the onset of Alzheimer's and as a neurosecretory product. Presently, the most common method to measure zinc involves (toxic) histochemical staining. An analytical PEBBLE sensor has been fabricated that incorporates two dyes, one is a reference and the other is sensitive to zinc, which enables the sensor to be ratiometric. This scheme is extendable to enzyme-based biosensing. The sensing components are incorporated into a polymer or a sol-gel matrix by either a microemulsion process or a modified sol-gel method. The nanosensors produced are in the size regime of 20–200 nm. Cellular measurements are made possible by the small sensor size and its biocompatibility. The effects of reversibility, photobleaching and leaching have been examined as well as the selectivity of zinc to other cellular ions such as $Na^+$, $K^+$, $Mg^{2+}$ and $Ca^{2+}$. Because of their fast response time, these sensors are capable of real-time inter and intra-cellular imaging.

### Real-time single-molecule study of cytokine–receptor interaction on living cells using nanoparticle probes

*Robert B. Jeffers and Xiaohong N. Xu, Old Dominion University, Chemistry & Biochemistry, 4541 Hampton Blvd, Norfolk, VA 23508, USA*

The function of cytokines as intercellular messenger molecules that initiate biological activity by binding with receptors on target cells is not well understood. We are particularly interested in the study of the dynamics of cytokine–receptor interactions on living cells in real-time. Little is known about these processes at the molecular level. Single-molecule analysis of cytokine function presents the potential for tracking and manipulating of individual steps in a sequence of biochemical events involved in the immune response. This understanding is essential to the design of immune-based therapies for vaccinations or to treat diseases. Nanoparticle probes were developed and employed to follow the real-time binding interactions of various cytokines with their receptors on living cells. These probes demonstrate the possibility for monitoring individual cytokine–receptor interactions on the surface of living cells. By following these interactions in real-time it is possible to monitor cellular response. Real-time analysis also allows for the determination of reaction kinetics, i.e. binding affinity, at the single-molecule level. The experimental configuration, updated research results and prospective applications will be discussed in detail.

### Calibration of mercury analysers and dynamic vapour generators

*Michael Markelov[1], John Kroczek[1], Joseph Siperstein[1], Sergey E. Sholupov[1], Nikolay R. Mashyanov[1] and Vladimir V. Ryzhov[2], [1]OhioLumex Co., R&D, 5405 E. Schaaf Road, Independence, OH 44131-1337, USA, [2]OhioLumex Co., Mercury Analytical System, Moskovskij Pr. 19, St Petersburg, Russia 198005*

Both static and dynamic methods can be used for preparation of mercury standards in air. Static methods call for the establishing of equilibrium between mercury metal and the confined vapour phase at constant temperature. These are the most accurate methods because they are based on the fundamental properties of matter and are independent of flow rates or geometrical shapes of vessels and samples. However, they can produce only limited amounts of vapours. The dynamic methods can produce practically infinite volumes of mercury vapours. However, their accuracy is always in question. Permeation tubes and similar devices do change their permeation rates with time and have all kinds of connecting tubing, valves, and joints where traces of mercury can be randomly lost. They are calibrated by observing their weight loss. This takes a very long time and there is no guarantee that weight loss is only due to loss of mercury and not the impurities in the polymeric membrane of the tube, especially, at the ng/M3 range. This paper will present the RA-915+ atomic absorption spectrophotometer with Zeeman correction, which can be calibrated using sealed mercury containing cells and flow through cells and to relate one to the other. The sealed cells can be placed directly in the optical path of the instrument avoiding any losses of mercury associated with vapour transfer. Therefore, it allows one accurately and quickly to determine the concentration of mercury in a flow through cell to calibrate dynamic vapour generators. The calibration approach using water-based standards will be discussed as well.

### Justifying the purchase of a laboratory information management system (LIMS)

*Howard J. Rosenberg, Thermo LabSystems, Sales, 100 Cummings Ctr, Beverly, MA 01915-6115, USA*

Justifying the purchase of a LIMS has been a topic that has received a great deal of attention since the institution of these automation systems. During the justification process, it is imperative initially to establish the complete cost of ownership. With the adoption of client server technology into this space and most recently with the provision of these systems under an Application Service Provider (ASP) paradigm, fully documenting the cost of ownership has become particularly critical. The justification process needs to take into account both quantitative and qualitative aspects. Within the quantitative portion of justification, it is essential to document traditional time/motion study results for a number of key personnel. It is important to include all personnel who will be accessing the LIMS as well as those who are dependent on the information derived from these systems. Of course, it is also beneficial to take into account any process cost savings that can be derived from the implementation of a LIMS, if applicable. Qualitative aspects, while not always easy to document are also a key element of preparing a complete justification for the purchase of a LIMS. This paper will detail the complete cost of ownership of a LIMS while pointing out several areas of 'hidden' costs that commonly overlooked. Both the quantitative and qualitative aspects of LIMS justification will then be discussed and explored with an eye towards discovering non-obvious cost savings. The data derived will then be applied to the purchase of a LIMS operating within a client server environment and then contrasted to a LIMS operating within the ASP model.

### Implementing LIMS and automation in an energy laboratory

*Don Kolva, Lisa Gorenflo and Christine Paszko, ATL, Inc., 496 Holly Grove School Road, West End, NC 27376-8412, USA*

Following an internal automation audit by a quality systems engineer at a fortune 500 firm of an analytical energy laboratory (gas and electric), several areas were targeted to provide automation and productivity enhancements. Following a systematic evaluation of the current laboratory processes, the following areas were identified to enhance turnaround time; sample login (automatic ID), instrument integration, reporting, (approval and transmitting results), and rapid result dissemination. Improvements that were implemented included, instituting bar coding and label generation to assist in streamlining sample login. This insured proper identification of samples throughout the laboratory and saved time over manual label generation. The decision was made to implement instrument integration to enhance data quality, reduce transcription errors and increase productivity. Reporting was enhanced in several ways. First, signatures of laboratory managers were digitized and placed on reports. Next, when managers viewed and approved results, the reports were automatically faxed from the LIMS to various clients, and hard copies were automatically printed. Finally, in order to rapidly disseminate information on any field samples (such as those for gas leaks) that exceed warning limits, the LIMS automatically sends e-mail to responsible managers and the cc list of other group members to act upon the results, the e-mail is forward to the managers cell phone for immediate action.

### Ensuring customer satisfaction with an LIMS customer relationship management module (CRM)

*Kim Skrecz and Christine Paszko, ATL, Inc., 496 Holly Grove School Road, West End, NC 27376-8412, USA*

As the economy slows and the business climate continues to become more competitive, firms that gain a reputation of excellent customer service and support have a strategic advantage over their competitors. Word quickly spreads and these firms can gain increased market share. This paper will focus on the implementation of an CRM module integrated with LIMS (Laboratory Information Management System). Regardless of the type of laboratory or the types of tests performed all laboratories receive complaints or inquiries on the testing performed or the final product. A water quality laboratory will be used as the model in this case study, but this module can be applied to any laboratory type. The laboratory receives calls on the aesthetics of the water quality, the taste and odour, the colour and other attributes. The ASTM (American Society of Testing and Materials) has a procedure for analysing water aesthetics and reporting on the flavour profile. The results of this testing can be entered into the database and users can follow the standard procedure for performing the testing. In addition to internal testing, users are also able to enter complainants received from customers, the client name and contact information, the nature of the complaint, the location, the data and time. With this information, a sample can be collected for analytical testing. The results of the testing are held in the LIMS database integrated with the CRM module so that results can be compared and investigated. For example, if a water utility customer reports a strong chlorine odour in their water, a sample may be collected, analysed and perhaps confirm high levels, there may be a problem with the distribution system. With this information in the LIMS, it is possible to investigate rapidly and possibly resolve the problem. Users can graph the results and monitor trends; print regular reports on open issues and assist managers is achieving customer satisfaction goals.

### Balance automation: in search of the ultimate solution

*Robert Pavlis and Steve Bolton, Labtronics, Inc., 546 Governors Road, Guelph, N., Canada N1K 1E3*

The balance is one of the most prevalent instruments in the world of analytical chemistry. Its wide spread usage in a vast array of analytical procedures generates tremendous amounts of data. Automation of the capture and processing of balance data is sure to increase accuracy, consistency, efficiency and productivity in any laboratory. Surprisingly, balances can be among the most difficult instruments to interface. Although the data are simple, compared with virtually any other instrument, most users normally want to do more than just transfer the numbers. Finding the right balance automation solution has more to do with the application and the user interaction than with the actual data. This presentation looks to the world of Chromatography Data Management as a model for the ultimate solution, one that will use client/server architecture to improve both the flexibility and the security of balance automation applications. This presentation unveils the world's first Balance Data System. A central server performs all of the processing, running many different procedures at the same time, servicing any number of clients and storing all of the collected data. Storing the data on the server increases security and assists in meeting regulatory requirements such as 21 CFR Part 11. Because the client simply acts as an interactive display, the client hardware can be any computing device, a desktop PC, a laptop or a PDA. The system lets users define their SOP, and then the control program carries out the SOP, stepping the user through each stage of the process. Controls can be added to check values, control editing, and even check the balance to ensure that the calibration schedule is followed. An SOP can be defined with specific hardware requirements and for specific hardware devices, so that if a five decimal balance is required, only suitable hardware can be used. The SOP can also be assigned on a user basis. When a user logs on to a system, they will only have access to the SOPs assigned to them. Capabilities for reporting to LIMS and getting work lists from LIMS are built in. This ultimate solution addresses application and user requirements in a cost-effective manner, dramatically increasing the potential for balance automation throughout the laboratory.

### The analysis of natural products by reversed-phase liquid chromatography using a novel bonded silica C18 column following accelerated solvent extraction

*Andrea W. Heckenberg[1], Rosanne Slingsby[1], Ruthann Kiser[1], Andrei Bordunov[1], Lyle Covino[2] and XiaoDong Liu[3], [1]Dionex Corporation, 1228 Titan Way, Sunnyvale, CA 94085-4074, USA, [2]Dionex Corporation, Salt Lake City Technical Center, 1515 W. 2200 S., Ste A, Salt Lake City, UT 84119-7209, USA, [3]Dionex Corporation, Three Biotech, 1 Innovation Drive, Worcester, MA 01605-4307, USA*

Consumer interest in natural products has exploded in recent years. The perceived benefits of these nutraceuticals covers a wide range, from relieving headaches to boosting the immune system. The therapeutic compounds that are responsible for these effects can be found in all parts of a plant, including the flowers, the leaves, the stems and the roots and include pharmacologically active compounds such as vasodilators, anti-oxidants, antibiotics and sedatives. Although this industry is not yet regulated, manufacturers of natural products are investigating analytical methods to produce and monitor consistent and clean product. Therefore, the separation and identification of natural products by HPLC is receiving much attention. To extract the compounds of interest, the plant is often pulverized manually in a volume of solvent and then filtered before injection. This adds considerable labour-intensive time to the total analysis time. An alternative and less labour-intensive method to extract the nutraceuticals is Accelerated Solvent Extraction (ASE), an extraction technique that speeds the extraction process and reduces the total amount of solvent required. The system uses conventional solvents at elevated temperature and pressures, which results in improved extraction kinetics. In this paper, novel bonded silica reversed-phase columns are used to characterize the composition of a series of nutraceuticals, following ASE extraction. These columns have been optimized for efficiency; hydrophobicity and low column bleed for use with mass spectrometers, for positive component identification.

### Semi-automatic sample deposition and its application to attenuated total reflection/Fourier transform infrared spectrometric analyses

*Jessica L. Jarman[1], Richard A. Todebush[2] and James A. de Haseth[2], [1]University of Georgia, 472 Creek Hollow Way, Athens, GA 30601-1491, USA, [2]University of Georgia, Department of Chemistry, Cedar Street, Athens, GA 30602, USA*

Fourier transform infrared (FT-IR) spectrometry is a useful tool for structural analysis. When microanalysis is required or desired, attenuated total reflection (ATR) elements may be used in FT-IR analyses to accommodate smaller sample quantities and lower concentrations. With the development of these sensitive and compact analysis systems, the need for automated sample deposition has arisen. Unfortunately, smaller sample volumes increase the difficulty of sample delivery. Characteristics of a reliable deposition system include reproducible results such as the total volume deposited and the placement of the deposits. Due to limited sample quantities, analyses are made more difficult because of sample manipulation that requires precise placement of minute volumes. These small volumes favour single bounce ATR techniques if spectral analysis is carried out. We have been successful in the development of a semi-automated liquid sample depositor for use with a single bounce ATR accessory, specifically a Harrick SplitPea. The depositor is fashioned from a series of valves and a glass nebulizer made to custom specifications. The system can be used to deposit small quantities reproducibly onto an area about 400 μm in diameter, which is ideal for the SplitPea. The internal reflection element has an active site of only about 250 μm in diameter. When comparisons between traditional manual deposits and semi-automated deposits are made, the semi-automated deposits are superior in deposit thickness, shape, and splatter characteristics. The design has been characterized for deposit size; precision of placement, and average deposit thickness. Additionally, variables such as sample volume and solvent elimination techniques can be easily altered. Due to this flexibility, the system is applicable to many analyses and sample types.

### On-line coupling of nuclear magnetic resonance to capillary electrophoresis: improved detection capabilities and extension to trace impurity analysis

*Andrew M. Wolters[1], Dimuthu A. Jayawickrama[1], Cynthia K. Larive[2] and Jonathan V. Sweedler[3], [1]University of Illinois at Urbana-Champaign, Chemistry, 600 S. Matthews Avenue, Urbana, IL 61801-3602, USA, [2]University of Kansas, Chemistry Department,* 2010 Malott Hall, Lawrence, KS 66045-7564, USA, *[3]University of Illinois at Urbana-Champaign, Department of Chemistry, 405 N. Matthews Avenue, Beckman I, Urbana, IL, USA*

Providing structural data unattainable by other techniques, nuclear magnetic resonance (NMR) spectroscopy represents an intriguing detector for capillary electrophoresis (CE). Wrapped around capillaries, microcoils, the best NMR coils in terms of mass sensitivity, afford easy on-line integration of NMR to CE. Recent improvements in coupling NMR to two different types of CE, capillary zone electrophoresis (CZE) and capillary isotachophoresis (cITP), are presented. Through high levels of sample stacking, cITP greatly increases the effective concentration sensitivity of microcoil NMR for charged species. With the added benefit of electrophoretic separation provided by cITP, this coupling offers a powerful means to analyse trace charged natural and synthetic organic impurities. If a charged trace impurity possesses a greater electrophoretic mobility than the main component, it can be efficiently isolated from the main component and simultaneously concentrated for NMR detection. As a demonstration, a 9.4 μl injection of 200 μm (1.9 nmole) atenolol, a charged pharmacological agent, obscured by a 1000-fold excess of sucrose (200 $m_M$) is analysed by cITP/NMR. By completely separating the atenolol from the sucrose and stacking it 200-fold (up to 40 $m_M$), cITP enables rapid $^1$H-NMR detection of the isolated trace component (10 s observation time). In comparison with the same microcoil probe operating without the benefit of sample stacking, cITP reduces experimental time by a factor of 40 000. For this particular probe and sample, greater stacking efficiencies than that achieved here would not improve NMR observation as most of the injected atenolol sample occupies the microcoil during the peak maximum. NMR probes consisting of multiple microcoils arranged in series facilitate loading and trapping of focused cITP bands for stopped-flow NMR analysis through the use of a scout coil. Using multiple coil technology in a slightly different manner, NMR probes consisting of multiple microcoils arranged in parallel can successfully avoid spectral degradation experienced during continuous-flow CZE/microcoil NMR. By cycling the electrophoretic flow from a separation channel between different outlets, high-resolution stopped-flow NMR spectra can be obtained from a continuous CZE separation.

### NMR prediction systems for pharmaceutical applications

*Gregory M. Banik, Eric Melanson, Victoria Rafalovsky and Refik Karabay, Bio-Rad Laboratories, Informatics Division, 3316 Spring Garden Street, Philadelphia, PA 19104-2552, USA*

A number of systems for prediction of $^{13}$C and $^1$H-NMR spectra are currently available. This paper reviews commercially available systems used in the characterization of compounds in drug discovery. Parameters impacting the quality of the predictions are enumerated, and the scope and limitations of each system are compared.

### Recent advances in the automated structure elucidation of a series of related natural products from the Cryptolepus family

*Antony J. Williams[1], Kirill Blinov[2], Eduard Martirosian[2], Mikhail Elyashberg[3] and Gary Martin[4], [1]Advanced Chemistry Development, 225 Jamestown Road, Pittsboro, NC 27312-6752, USA, [2]ACD, Development Office, 90 Adelaide Street West, Toronto, ONT, Canada M5H 3V9, [3]All-Russian Research Institute of Organic Synthesis, Radio St 12, Moscow, Russia 107005, [4]Pharmacia, Pharmaceutic MS 4821 259, 7000 Portage Road, Kalamazoo, MI, USA*

Significant literature exists about the elucidation of a number of related natural products from the Cryptolepus family. This literature has allowed us to test recent developments in our automated structure elucidation development. Our previous work has used an expert system based on a database of $^{13}$C-NMR spectra and a library of molecular fragments and their $^{13}$C-NMR subspectra. We have recently implemented the ability to use information obtained from 2D spectroscopy including direct and long-range heteronuclear correlations for both C13 and N15 nuclear centres. The PC-based software solution presently delivers the ability to process raw 1D and 2D data, elucidate possible structures using the fragment databases, predict the $^1$H- and $^{13}$C-NMR spectra of the potential structures for comparison and train the prediction databases in order to use fragments from related structures for the automated elucidation process. We will review recent developments and successes in the application of automated elucidation to natural products, which in some cases have reduced the manual task of elucidation from many days to just a few minutes.

### High-speed GPC analysis, column design, performance and application

*Daniela Held, Günter Reinhold and Thorsten Hofe, PSS Polymer Standards Service, PO Box 3368, Mainz D-55023, Germany*

SEC is the most used technique to characterize polymers. The most important items in SEC instruments are the columns for separation. PSS introduced HighSpeed SEC columns in 1999 which are able to separate all kinds of polymers in < 2 min, cutting down the time requirements of traditional SEC runs by a factor of five to 10. The ability to generate a 12-point calibration curve in 10 min is unparalleled. With the availability of such high-speed columns SEC results can be given extremely fast, which is necessary on one hand for in-line production control systems (quality control) and high-throughput screening devices and on the other hand for high-end applications like 2D chromatography. Compared with their analytical counterpart (8 × 300 mm) HighSpeed columns have larger diameters and a smaller length (20 × 50 mm). Therefore, they permit higher flow rates with equal linear flow, equal resolution and polymer information. The flow rate is more limited by instrumentation (e.g. detectors, pumps) than by the column itself. The HighSpeed columns are studied concerning performance (monodisperse and polydisperse samples), accuracy and reproducibility in aqueous and organic solutions. The results obtained in just 20% of the time are comparable to analytical columns. Low shear forces on the HighSpeed columns result in less stress for the characterized samples, which is important for high molecular weight products. Nevertheless, there is still room for further SEC column development. It requires the skill and dedication of the chemists who create the packing materials and the profound understanding of the underlying principles and applications.

### Keeping archived data active

*Michael Elliott, Scientific Software, Inc., 6612 Owens Drive, Pleasanton, CA 94588-3334, USA*

Archiving data for increased security and/or long-term storage does not have to mean that the data are no longer an integral part of a scientist's daily work. Finding new meaning in old data or relationships between new and old data can save a significant amount of research and development time. The limited disk space of some permanent storage media and the high cost of read/write devices often mean that archived data must be stored off-line. This paper will describe a software package that makes archived data actively available for searching, viewing and retrieving to users, whether stored on-line or off-line. The physical transfer of data from active to archive is an important step in the life cycle of any company's records. This transfer of custody does not have to mean that the data ceases to be useful. CyberLAB Knowledge Engineering System, from Scientific Software, makes archived records available whether stored on-line, near-line, or off-line. When electronic records are uploaded into the CyberLAB system, keywords, text and results values can be extracted and stored as part of the record's metadata. These metadata can be search through any of CyberLAB's search mechanisms. During the archive process, these metadata are exported as an XML file and is archived with the data, however the database retains a copy of this metadata, so the data file remains available for searching. When searching for data with any of CyberLAB's search tools, both archived and non-archived data can be returned. Even when the data have been stored on removable archive media, such as CD or DVD, and been removed from the system, the metadata can be searched. The only indication that the end user has that the data has been archived is a small 'lock' icon next to the file name. CyberLAB does not alter the original file during the archive process. This means that an archived file stored on-line can be viewed immediately simply by clicking on the file link. Should the file be stored off-line, the user has the option to send an e-mail request to the archive administrator to load the required archive volume. With this 'active archive' feature researchers can search for new meaning in previous work, or correlate preceding studies with current.

### Automating data management

*Soheil Saadat, Scientific Software, Inc., 6612 Owens Drive, Pleasanton, CA 94588-3334, USA*

The increasing automation of laboratory instrumentation and processes has led to a substantial increase in the amount of data produced. This data must be transformed into meaningful information as quickly and efficiently as possible. Extracting more knowledge from existing information without overburdening scientists depends on the power of the automation method. This paper describes an automation method for the knowledge engineering process. To be an effective automation tool, this system must monitor the entire enterprise for new or altered data to be uploaded into the system—no user interaction should be required. This software system can monitor specific computers, drives and/or folders for general or very specific files to transfer. Once maintained and protected inside the software, files can be monitored for automated archival based on time schedule or file age. A variety of archive devices and archive schedules can be supported simultaneously. On file upload, options must be available for automated metadata extraction whether results values or searchable text. This information can be extracted from files as they are transferred and is available for searching immediately. During the lifetime of any piece of data, the information needs will change. It is essential that users be able to update a file's metadata with additional information or searchable keys quickly and easily. Once the data has been added to the system users must be able to retrieve it in such a way that new relationships between data can be discovered and explored. Simple keyword searches can produced lists of files related by content. Advanced search features allow deeper similarities between different data types to emerge. Both simple and complex searching mechanisms are crucial components to effective data mining.

### Software quality assurance through the proper methodology

*Tim Meehan, Todd Stewart and Joel Hunter, RTS Enabling Technology, 3200 George Washington Way, Ste D, Richland, WA 99352-1664, USA*

The Standish Group Report determined that the success of software projects is incredibly low; whereby 31% of all software projects are cancelled before completion, 53% of projects will cost 189% of estimates, 9% on time and on budget (large companies), and 16% on time and on budget (small companies). Success was gauged on restarts of projects, cost overruns, time overruns and percentage of functionality. The high rate of failure was largely attributable to lack of user input, insufficient requirements and changing requirements. With the demands of the Internet as well as federal regulations from the FDA and DOE, quality assurance in software is increasingly becoming the main focus of software development. To mitigate the failures of past software development, a number of methodologies have arisen to address these issues. This seminar will discuss two of these methodologies, which are gaining considerable favour in the software industry: the Rational Unified Process and Xtreme programming. The similarities and differences of the two methodologies will be discussed, and how they fit the needs of the software owners and the software developers.

### Laboratory QA/QC—the benefits should be obvious

*Alan M. Czyzewicz and Daniel E. Meils, Scientech, Inc., O&M, 2650 McCormick Drive, Clearwater, FL 33759-1005, USA*

This paper presents a viable approach to ensuring an appropriate degree of confidence to the precision and accuracy of the data. Thereby satisfying customer's requirements, minimizing rework, inappropriate calibrations, maintenance and repairs. When you know and understand your customer's valid requirements for analyses, Statistical Process Control (SPC) becomes the tool to lower costs. SPC allows for management by exception, thus focusing on work where it is needed. Shewhart control charts are needed to be at the foundation of SPC. To benefit most from the appropriate chart, a methodical approach to its development is needed. Twenty to 30 data points from an appropriate sampling of technicians and conditions are at the foundation. Statistical tests for outliers, normality of data and % RSD need to be calculated and examined. Bias should be determined with respect to the target value and centreline adjusted accordingly. Once the chart has been developed, key indicators must be examined with each QC analysis. Finally, long-term trends should be examined via SPC by reviewing bias with respect to target value, change in precision and normality of data. Benefit One Satisfy customer requirements by quantifying accuracy and precision through statistical analyses of your methods. Customers can chose the methods that yield the most appropriate accuracy and precision to meet their needs and budget. Benefit Two Minimize rework by using SPC to indicate when the analysis is Out of Control (OOC) before the actual performance of analysis. Minimizing reanalysis produces costs savings. Benefit Three Minimize inappropriate calibrations, maintenance and repairs by using SPC to determine when an instrument is OOC and corrective actions are required. 'Don't fix what ain't broke.' Statistical programs, such as Lab Stats Pack$^{TM}$, are available to perform these calculations and reviews. Clearly, starting with the knowledge of your customer's valid requirements and managing your instrumentation by statistical exception will create obvious benefits to your laboratory QA/QC program.

### Scientific information production

*Kim Ahmed and Malcolm McGregor, LabVantage Solutions, 245 Highway 22, Bridgewater, NJ 08807-2560, USA*

Traditionally science has been viewed as a handcrafted occupation. Skilled scientists are employed to plan and undertake experiments and report on their findings. In academia, the end result is scientific papers and patents and in industry Quality reports or patents during the research phase. Modern discovery oriented sciences have made these methodologies as obsolete as Ford made the hand-built automobile. There are two fundamental aspects of discovery activities that have forced the changes. First, the volume of data being produced is overwhelmingly beyond a human's ability to reduce to meaningful information without computerized automation and, second, in many instances it is the information itself that is the 'product' of the laboratory. It could therefore be said that discovery laboratories are in the business of 'Scientific Information Production' and should view their processes, workforce and product accordingly. This paper examines the proposition that 'Scientific Information Production' is a valid model for these laboratories. The paper goes on to identify the pieces of automation required to achieve the necessary efficiencies and finally to look at a software model designed to unify these disparate entities into a true production system. Each phase of the production environment is examined from customer interaction and experimental design through operations and data collection to packaging the final data for sale or use. Key obstacles from the past such as the integration of existing expert systems and the portability and compatibility of various data formats are given special attention.

### Specialty gas session: a fast, innovative infrared analyser for monitoring ultratrace moisture in semiconductor FAB gases

*Wen-Bin Yan, Tiger Optics, LLC, 250 Titus Avenue, Warrington, PA 18976-2426, USA*

Evidence of direct correlation between gas purity and process defects has been documented in fields such as silicon epitaxy, oxidation and annealing treatments, and film deposition. This has driven equipment suppliers to develop new analytical detection systems for monitoring ultratrace gas impurities in bulk and specialty gases for the semiconductor fab. Moisture is a major contaminant in the semiconductor fab gas system. A trace gas analyser, the MTO-1000 has the detection capability to measure to 500 PPT moisture and expand to monitor for PPB or sub-PPB for other trace gas impurities such as ammonia, an airborne molecular contaminant that must be constantly monitored at ultratrace levels. The MTO-1000 uses a small, inexpensive diode laser that can operate in various parts of the infrared spectrum. The principle of operation is Cavity Ring-Down Spectroscopy (CRDS), which was jointly developed by Tiger Optics and Princeton University. In the gas phase, the spectrum of a molecular species consists of many sharp rotational lines that provide high selectivity for detection. This is the 'molecular fingerprint' of that species. The diode laser provides the narrow bandwidth to focus on the major absorption lines for high accuracy and maximum absorption of a trace gas impurity. Data from gas suppliers will be presented and discussed on fast, trace gas impurity detection of trace moisture in both inert and corrosive gases.

### Specialty gas analysis: analysis of trace-level moisture in corrosive and reactive gases using an evacuated FTIR gas analyser

*Robert Klebba and Peter Zemek, Midac Corporation, 17911 Fitch, Irvine, CA 92614-6016, USA*

The demand for increasing purity in semiconductor gases is pushing the allowable moisture levels in these gases $< 1$ ppm and down to the low ppb level. For inert gases, there are relatively simple technologies for directly detecting moisture down to single-digit ppb concentrations. However, these techniques cannot be used for corrosive or otherwise reactive gases such as hydrogen bromide (HBr) or ammonia ($NH_3$). Fourier-transform infrared spectroscopy has been used for analysing moisture and other contaminants in semiconductor gases for many years. Gas cells made of corrosion-resistant materials isolate the gas from the spectrometer components, allowing easy purity and concentration analysis. High-resolution FTIR spectrometers overcome problems due to spectral interference, and FTIR's inherent photometric accuracy provides long-term calibration stability. Early attempts at FTIR moisture analysis were plagued with problems due to the unstable concentration of moisture inside the analyser. Even with UHP nitrogen purge, the analyser would take days to dry down, or leaks might occur allowing the infiltration of ambient moisture. These and other factors kept earlier analysers from achieving > 500 ppb limits of detection. A different approach involves removing all of the moisture inside the spectrometer by evacuating down to $10^{-6}$ torr. This level of vacuum effectively removes all moisture inside the spectrometer, so that only the gas analyser detects the moisture in the semiconductor gas. In this presentation, we will discuss the special considerations for an evacuated FTIR gas analyser as well as the selection of specific components to optimize performance for moisture analysis.

### Specialty gas session: temperature dependent infrared absorptivity data to support stack gas emission monitoring

*Pamela M. Chu*[1], *George C. Rhoderick*[1] *and Patricia A. Johnson*[2], [1]*NIST, Analytical Chemistry Division, Bldg 235 B117, Gaithersburg, MD* 20899-0001, USA, [2]*NIST, 20 Chatfield Pl. E., Painted Post, NY 14870-9361, USA*

The infrared absorptivity of industrially relevant compounds methanol and sulphur dioxide are evaluated for temperatures ranging from 25 to 200 °C at 25 °C increments using an FT spectrometer with NIST primary gravimetric standards. This project was initiated to provide quality assured reference data for communities measuring stack emissions by passive FT-IR and other infrared-based technologies. To correct for the sample and sample cell emission effects, sample and reference data are acquired with the infrared source on and with the infrared source off. The average relative expanded uncertainty for the uncorrected absorption coefficients is 2.0%. The emission correction tracks the blackbody emission of the sample cell and is both wavelength and temperature dependent. For temperatures up to 200 °C, there is no emission correction for bands at $\geq$2600 cm$^{-1}$. In the finger print region, the emission correction be-comes significant especially at temperatures > 100 °C. At 200 °C, the correction is approximately 10% for bands in the spectral region near 1000 cm$^{-1}$. To validate further the absorption coefficient data, the spectra are compared with available data and models.

### Monitoring proteins in real-time and in homogeneous solution

*Weihong Tan, University of Florida, Department of Chemistry & The McKnight, PO Box 117200, Leigh Hal, Gainesville, FL 32611-7200, USA*

As the Human Genome is completing, researchers are turning increasingly to the task of converting the DNA sequences into information that will be highly useful in human medicine and healthcare. One of the key challenges ahead is to understand proteomics, the science of the cellular protein universe. It is expected that in the post-genome era, proteomics and the study of protein functions will play an increasingly important role in the understanding, diagnosis and treatment of the most challenging human diseases such as cancer, AIDS and heart disease. The development of new molecular probes for protein analysis is of great interest. We have developed novel protein recognition mechanisms for real-time protein monitoring using molecular beacons (MB) and molecular beacon aptamers (MBA). MBs are a new class of oligonucleotides that can report the presence of specific nucleic acids. They have an excellent signal transduction mechanism. Molecular beacon aptamer is developed by combining MB's excellent signal transduction and aptamer's protein affinity for real-time protein recognition with extremely high sensitivity and excellent specificity. We will report our progress in protein detection using MBAs. These proteins are important biomarkers for cancer diagnosis.

### Breaking the speed limit in planar chromatography

*Huba Kalasz, Semmelweis University, Department of Pharmacology, Nagyvarad Ter 4, Budapest H-1089, Hungary*

Each type of separation methods has limited speed. Both gas chromatography and liquid chromatography can be optimized according to the flow velocity of the mobile phase. A definite optimum can generally be found which is determined by certain factors such as the eddy diffusion, mass transfer and longitudinal diffusion. Planarchromatography takes a special position among the separation techniques. The classical mode of thin-layer chromatography (TLC) is realized on stationary phase, which is dry (non-wetted) at the beginning of the separation. There is a gradient of the mobile phase to the stationary phase ratio that is increasing during the development. FF-TLC (forced-flow thin-layer chromatography) kept the majority of features of TLC, and an essential speed-up of the mobile phase flow velocity was given by the forced-flow. Also, the optimization of flow velocity of the mobile phase was possible by the constant and regulated mobile phase supply. The optimum flow velocity of the mobile phase was checked, and the plate high of the system at optimum flow velocity was near to the particle diameter. Planar electrochromatography (PEC) is the technique of great promise. As the eddy diffusion is practically eliminated in PEC, it means really breaking the speed limit in planar chromatography. Separations may be performed in 10 to 100s of seconds instead of several to 10 min. PEC can be performed using a wide variety of stationary phases. The stationary phase is wetted before the PEC, thereby closing the electric circuit. The choice of the mobile phase depends on the solutes to be separated. Aqueous mobile phases generate higher current; therefore, a cooling system keeps the temperature constant. PEC in organic solvents generates low current, and both the solvent resistance of the set-up and thermostating at a constant temperature can be offered by the adequate construction of the system, which is made of glass. This project as sponsored by the grants of OTKA T025142, T032185 and T034677.

### High-speed separation of biopolymers in microfabricated devices

*Andras Guttman, Torrey Mesa Research Institute, 3115 Merryfield Row, Suite 191, San Diego, CA 92121-1125, USA*

Rapid and large-scale genotyping, mapping and expression profiling require affordable, fully automated high-throughput devices enabling rapid, high-performance analysis using minute quantities of reagents. Electric field-mediated separation methods in capillary dimensions, such as CE, ultrathin-layer electrophoresis and microchip-based techniques have been emerged in recent years to address rapid analysis of biologically important molecules, such as nucleic acids and proteins. These novel, high-performance separation techniques feature rapid separation times and very high resolving power, while requiring only very little amounts of samples and reagents. The applicability of electric field-mediated microseparation methods in capillary dimensions will be discussed for rapid and high-resolution analytical and micropreparative separation of biomolecules, using replaceable polymers as separation matrices. Various ways of automation for large-scale analysis of biological samples (96-well plate format) will also be demonstrated. While electric field-mediated separations in capillary dimensions are primarily considered as analytical tools, they can also be used in micropreparative applications. In micropreparative operation mode, nanomolar quantities of DNA fragments were collected for subsequent downstream processing, such as sequencing for rapid expressed sequence tag (EST) generation to establish unigene sets and for high-throughput cloning.

### Determination of free aluminium in aqueous media by flow injection liquid–liquid extraction and graphite furnace atomic absorption spectrometry

*Julian F. Tyson[1] and Ahmed Youssef[2], [1]University of Massachusetts, Department of Chemistry, 710 N. Pleasant Street, Amherst, MA 01003-9305, USA, [2]Cairo University, Chemistry, Faculty of Sciences, Giza, Egypt*

We have developed a procedure for the speciation of aluminium in natural water systems based on the reaction of 'free' aluminium with 8-hydroxyquinoline and extraction of the resulting complex into isobutlymethyl ketone (IBMK). To restrict the extracted aluminium to that which was only able to react rapidly, the kinetics of the formation and extraction processes were controlled in a flow-injection system. To determine the low concentrations likely in natural waters, quantification was by graphite furnace atomic absorption spectrometry. The problem of pumping the IBMK was overcome by the injection of a relatively large volume (2 ml) into an air carrier stream. The system contained a novel-phase separator device, which was fabricated in-house based on a design of Zhaloun Fang's. This device has a conical Teflon cavity mounted on a stainless steel base; the IBMK wets the Teflon and is drawn out via an exit at the apex of the cone, whereas the aqueous phase wets the stainless steel. The system was evaluated by comparing the measured fraction of free aluminium with that calculated from the relevant equilibrium constants for several model ligands (EDTA, oxalate and malonate). Good agreement was obtained. The procedure was applied to the study of the aluminium binding to a humic acid material, the aluminium speciation in surface water, and to a study of the kinetics of dissociation of aluminium complexes and polymeric aluminium species. The procedure was applicable to concentrations down to 2 ppb. The surface water contained 60 ppb of dissolved aluminium of which 2.4 ppb was reactive. On acidification, 20 h was needed to release the aluminium bound to the humic acid material.

### Determination of antimony by flow injection hydride generation atomic absorption spectrometry after photo-oxidative sample pretreatment

*David J. Scott and Julian F. Tyson, University of Massachusetts, Department of Chemistry, 710 N. Pleasant Street, Amherst, MA 01003-9305, USA*

Increased analyte transport and the removal of interfering matrix effects are two advantages of hydride generation (HG) sample introduction in atomic spectrometry compared with standard nebulization. HG is widely used in the trace determination of arsenic, selenium, mercury and antimony with various atomic spectrometry detectors. As toxicity is directly related to chemical species, the trend in research has been towards speciation based on chemical or chromatographic separation with subsequent element-specific detection. Recent reports describe the speciation of inorganic antimony by manipulation of the oxidation states before hydride generation, although the measurement of total antimony content with little regard to speciation is often reported. There is currently interest in organoantimony [1] compounds, such as trimethylantimony, found in urine [2]. The hydride generation chemistry of trimethylantimony has been the subject of discussion due to the demethylation [3,4] of the hydride species and subsequent molecular rearrangement resulting in broad peaks and poor detection limits. One way to overcome these problems would be to convert the antimony species separated chromatographically to SbIII by a suitable post-column reaction before forming stibine. Here we present our investigations into the photo-oxidation of antimony compounds before reduction to SbIII and subsequent hydride generation.

1. Zheng, J., Takeda, A. and Furuta, N. *Journal of Analytical Atomic Spectrometry*, **16** (2001), 62.
2. Krachler, M. and Emons, H. *Journal of Analytical Atomic Spectrometry*, **16** (2001), 20.
3. Craig, P. J., Forster, S. N., Jenkins, R. O. and Miller, D. *Analyst*, **124** (1999), 1243.
4. Krachler, M. and Emons, H. *Journal of Analytical Atomic Spectrometry*, **15** (2000), 281.

### Atomic absorption determination of inorganic and methylmercury by flow injection chemical vapour generation and amalgam trapping

*Julian F. Tyson*[1], *Christopher D. Palmer*[2] *and Susana Rio-Segade*[3], [1]*University of Massachusetts, Department of Chemistry, 710 N. Pleasant Street, Amherst, MA 01003-9305, USA,* [2]*University of Massachusetts, Empire State Plaza, Albany, NY 12223, USA,* [3]*University of Vigo, Chemistry, Lagoas-Marcosende, Vigo E-36200, Spain*

Studies of biogeochemical transformations are underpinned by the provision of reliable information about the chemical composition of relevant materials with respect to inorganic and organomoetallic constituents. With regard to mercury, while a number of organomercury compounds are known, it is the pathways involving inorganic mercury (i-Hg) and methylmercury (m-Hg) that are of current interest. We have been developing a procedure based on the reaction of the two species, i-Hg and m-Hg, with borohydride [1]. Our hypothesis is that the species form elemental mercury (e-Hg) and methylmercury hydride (m-HgH), respectively (though this is contrary to some published results, but in agreement with others). As we think that m-HgH does not absorb radiation from a Hg hollow cathode lamp, the direct transfer of the vapours generated to an atom cell allows determination of i-Hg only. As both species are trapped by gold–platinum gauze, both are determined via the amalgam trapping route. On this basis, we have accurately quantified the mercury species in some marine tissue reference materials from the National Research Council of Canada. Recently we have found that the extent to which e-Hg is produced from m-Hg is related to both the borohydride and the sodium hydroxide (added to slow the decomposition of the reagent in aqueous solution) concentrations, so that conditions may be selected under which both species form e-Hg. This opens a new possibility for a speciation method based on different borohydride/hydroxide concentrations. Selectivity is also obtained by the judicious use of hydrochloric acid to extract the mercury species from the samples. At low HCl concentrations, only m-Hg is extracted. Samples may also be handled as slurries in the FI system, and we have obtained selective preconcentration by sorbent extraction on C-18 following complexation with APDC. Detection limits were improved by up to a factor of 10.

1. Palmer, C. D., Tyson, J. F. and Schlemmer, G., Abstract 1359, Pittcon 2001.

### Real-time direct analysis of mercury in air using portable atomic absorbtion spectrometer with Zeeman correction

*Michael Markelov, Joseph Siperstein, Olga Bershevits, John Kroczek and David De Chant, OhioLumex Co., R&D, 5405 E. Schaaf Road, Independence, OH 44131-1337, USA*

This paper will demonstrate the direct analysis of mercury in air without any preconcentration, amalgamation, or trapping options generating real-time mercury values using the Atomic Absorption Spectrometer RA-915+ with Zeeman correction. This instrument was originally designed as a portable instrument to pinpoint the sources of mercury spills and accumulations. The high sensitivity of the instrument (several $ng\,m^{-3}$) is provided by a built in 10-m multipass cell. The selectivity of the instrument to mercury is assured by using a single mercury isotope lamp along with Zeeman correction. To address high levels of mercury contamination, the instrument is also equipped with a 6-cm single-pass cell. The instrument also has a built in cell for instant QC check in the field. It can be operated via its own hand held controller/reader or using a notebook PC. The paper will also discuss approaches to calibration of Mercury analysers for analysis of air, water, soils and petroleum fractions.

### Real-time *in vivo* study of single-molecular biological events of bid

*Qian Wan*[1] *and X. Nancy Xu*[2], [1]*Old Dominion University, Chemistry & Biochemistry, 4541 Hampton Blvd, Norfolk, VA 23508, USA,* [2]*Old Dominion University, Spectroscopy and Raman Systems, 111 Downey Street, Norwood, MA 02062-2612, USA*

Bid is one of the widely studied apoptotic proteins. Upon the induction of apoptosis, bid will be cleaved into two parts: 6.5 kD tn-bid and 15 kD tc-bid. Previous studies have showed tn-bid bound tc-bid *in vitro* using Western blot and tc-bid moved into mitochondria to promote apoptosis. However, it is unclear whether tn-bid and tc-bid will bind in living cell and where tn-bid is located inside the cells. We study these cascades using real-time single-molecule chemical microscopy. This research aims to demonstrate the first single-molecule measurements of sequencing events *in vivo* and uncover the roles of bid in apoptosis *in vivo*. The tn-bid and tc-bid genes are cloned using plasmids and fused with red fluorescence protein and green fluorescence protein, respectively. Then, the plasmids are transfected into human cells and monitored in real-time using single-molecule dynamics microscopy.

### Application of near infrared spectroscopy in monitoring microbial and mammalian bioprocesses

*S. Alison Arnold, John Crowley, Linda M. Harvey and Brian McNeil, Strathclyde Fermentation Centre, Department of Bioscience, Glasgow G1 1XW, UK*

The use of Near Infrared Spectroscopy (NIRS) to monitor both microbial and mammalian cultivations was investigated. NIRS offers a number of advantages over other monitoring techniques in that it is fast, requires no pretreatment of sample, can monitor several analytes simultaneously and has the capability to be used on-line or *in-situ*; therefore, enabling analysis of both chemical and biological parameters in terms of real-time. Potentially, a real-time multi-analyte monitoring system could be very useful within the bioprocessing industry. In the present study, application of the technology was first investigated at-line (where the analyser is near the bioreactor although not in direct contact with it). The cultures investigated included *Escherichia coli*, *Streptomyces fradiae* and *Pichia pastoris*. Successful models were built and validated for a number of critical analytes and products within these three very different bioprocess fluids [1, 2]. Following the successful application of NIRS at-line the use of *in-situ* NIRS was examined. This was carried out using steam sterilizable fibreoptic probes in the bioreactor. Here the probe is in direct contact with the process stream inside the bioreactor allowing real-time analysis of analytes. Successful on-line models for biomass in a fed-batch *E. coli* system and glucose, glutamine, ammonia and lactate for a CHO-K1 cultivation were built and validated. The key issues in conversion from at-line to *in-situ* operation were discussed. The success of NIRS both at-line and on-line in monitoring such an array of different analytes from very varying bioprocesses bodes well for its routine use in an industrial setting.

1. Arnold *et al. Enzyme Micro Technology*, 27 (2000), 691.
2. Arnold *et al. Biotechnology Letters*, 23 (2001), 143.

### Material ID discriminator: towards an automated discriminator software algorithm

*Donald Kuehl[1], Hector Casal[2] and Dave Cameron[2], [1]Thermo Galactic, 395 Main Street, Salem, NH 03079-2464, USA, [2]DHC Analysis, 3645 Warrensville Center Road, Cleveland, OH 44122-5247, USA*

As we use the term, a discriminator is a algorithm that can be used to classify a spectrum within defined sets of spectra. Each set is for a given chemical composition: a pure material or a mixture. It can be used to confirm the identity of a material or automatically to identify a material. This concept is substantially different from that of a search algorithm. In a search, the goal is to identify the most likely spectral match, but confirmation of identity is done visually. Searches are not set up as discriminators, if they were then matches would seldom be obtained because of sample impurities and variations in spectrometers and spectral accessories. The greatest hindrance to setting up a discrimination package for a plant has been the lack of automation, with days or weeks of work to set up discriminators for the individual materials. This algorithm uses two variables for developing discrimination criteria from spectral data: a similarity parameter and a PCA model. The similarity parameter is calculated between a measured spectrum and a representative spectrum of each target material. The PCA models are used to confirm the results. To develop the discriminator model the user is only required to enter the name of the material and the spectra of a series of measurements. The program develops the models with no user input, appropriate masks are calculated for each spectral region and inappropriate spectra are identified. In use the program automatically classifies the spectrum based on the appropriate criteria, which are set automatically when building the models. Examples of the use of this package with several data sets will be presented.

### Applying equality constraints during alternating least-squares factor analysis

*Mark H. Van Benthem, Michael R. Keenan and David M. Haaland, Sandia National Laboratories, PO Box 5800, Albuquerque, NM 87185-0886, USA*

The use of constraints during multivariate curve resolution is critical to ensure one obtains a reasonable and physically meaningful solution. Typical constraints are non-negativity, unimodality, closure and selectivity. Frequently, equality constraints are employed to ensure that a variable, whose value is known or well defined, is contained in the solution. A common practice to employ equality constraints, presumably for computational convenience, is to solve the least-squares problem without constraining the solution, followed by replacement of the constrained variables with their known values. While this method is easy to program and fast to implement, it most often destroys the least-squares result. We will present established methods of solving the constrained least-squares problem. We will also discuss the benefits of using these methods and demonstrate how they can be employed in alternating least-squares factor analysis.

Sandia is a multiprogramme laboratory operated by Sandia Corporation, a Lockheed Martin Company, for the US Department of Energy under Contract DE-ACO4-94AL85000.

### Automated real-time determination of bromate in drinking water using IC-ICP-MS and proposed EPA Method 321.8

*Thomas Joseph Gluodenis[1], Jason A. Day[2], Anne Vonderheide[2] and Joseph A. Caruso[2], [1]Agilent Technologies, 2850 Centerville Road, Wilmington, DE* 19808-1610, USA, [2]*University of Cincinnati, Department of Chemistry, PO Box 0172, Cincinnati, OH 45221-0172, USA*

The suitability of coupling an HPLC to an ICP-MS for the fully automated, routine analysis of bromate in drinking water as per the proposed EPA Method 321.8 was investigated. The necessity to monitor the carcinogen bromate in ozonated drinking waters at single ppb levels has led the USEPA to investigate HPLC-ICP-MS as an alternative technique to the ion chromatography with conductivity detection method currently specified. During this investigation, a series of rigorous performance checks were used to assess the implementation of the proposed method including determination of the abundance sensitivity of the ICP mass spectrometer and the method detection limit, assessment of potential chromatographic interferences, and analysis of laboratory fortified blank and matrix samples. This was followed by the determination of bromate in a series of EPA disinfection by-product (DBP) standards. A description of the protocol used and the corresponding analytical figures of merit will be presented.

### A consensus method for available cyanide using ligand displacement and flow injection analysis amperometry

*Scott W. Stieg[1], Ninglan Liao[1] and John R. Sebroski[2], [1]Lachat Instruments, 6645 W. Mill Road, Milwaukee, WI 53218-1238, USA, [2]Bayer Corporation, State Route 2, New Martinsville, WV 26159, USA*

A new ASTM standard test method for available cyanide is described. ASTM Committee D19, Water Quality, developed this method. ‘Available Cyanide’ has been defined by the USEPA to replace or more precisely define the older term 'cyanides amenable to chlorination'. It includes the species HCN, $CN^-$, and a set of metal–cyanide complexes, such as mercury and nickel cyanides, that are easily dissociated into free cyanide ions. The method does not detect the less toxic strong metal–cyanide complexes such as ferrocyanide and ferricyanide. A proprietary version of this test method (OIA-1677) was approved by the USEPA in December 1999 for NPDES and NPDWR compliance monitoring. This ASTM method will be a consensus version of this method, and the collaborative study will lead to approval for these Clean Water Act and Safe Drinking Water Act programs. The apparatus is commercially available from both Lachat Instruments and OI Corp. A water sample is digested by batch addition of ligand-exchange reagents tetraethylenepentamine and dithizone solutions at room temperature. After a few minutes, the digested sample is injected into a carrier or donor stream which is acidified. The resulting HCN(g) diffuses across a hydrophobic membrane into an alkaline acceptor stream. This eliminates non-volatile interferents such as thiocyanate. The cyanide anion in the acceptor stream is detected at a silver electrode by oxidation of silver to silver cyanide and measurement of the resulting amperometric current at about 0 V versus Ag/AgCl. The amperometric method eliminates the pyridine used in the colorimetric version of this method. Weak acid dissociable (WAD) cyanide can also be determined by omitting the digestion with ligand-exchange reagents. As required by ASTM and the USEPA a collaborative study organized by the Bayer Corporation Environmental Testing Services was carried out by ASTM D19 during 2001–2002. The results of this interlaboratory study will be presented.

### Discrete analyser technology applied to environmental analysis

*Stephen C. Coverly[1], Joseph R. Jadamec[2] and Dan Aven[3], [1]Bran & Luebbe, Werkstrasse 4, Norderstedt D-22844, Germany, [2]University of Connecticut, Coastal Environmental Research F, 1084 Shennecossett Road, Groton, CT 06340-6048, USA, [3]Westo, 12 Precision Road, Danbury, CT 06810-7317, USA*

In clinical chemistry, continuous-flow (CFA) analysers have been almost completely replaced by 'discrete' analysers where each reaction takes place in a separate vessel. Discrete analysers provide more reliable analysis at lower cost and with lower manpower than CFA systems. However, the clinical analysers have found very limited application for water, food and industrial samples. One reason is that the requirements for accuracy and detection limit in clinical analysis are less rigorous than for industrial samples: another is the difference in techniques, colorimetric end-point analysis being the only technique widely applied to both fields. Furthermore, up to now no methods were available based on EPA approved chemistries. We have built a discrete analyser specially designed for environmental samples. Compared with a clinical analyser it has changes to the detector, sample and reagent holders and to the software. We have developed special hardware and software to automate the Cd reduction stage required by the EPA for nitrate analysis. All the 11 EPA water analysis methods commonly run with CFA have been automated. Reproducibility is > 1% RSD for each method, with detection limits (MDL) in the low $g\,\mu l^{-1}$ region. The analysers can automatically rerun samples in different ranges, with manual or automatic range selection. Sampling rate is method-dependent, and is 100–200 tests per h for most methods. We present comparison data obtained on laboratory quality control and actual environmental samples of fresh, brackish, and waste-water.

### A new approach to fast total organic carbon (TOC) analysis for process applications

*Reno Cerra[1] and Mike Retzik[2], [1]SGE International Pty Ltd, 7 Argent Place, Ringwood, Victoria 3134, Australia, [2]Anatel Corporation, 2200 Central Avenue, Boulder, CO 80301-2859, USA*

A Total Organic Carbon (TOC) analyser using a unique design will be discussed. The benefits of this newly development method include the ability to achieve fast TOC analysis with extremely low running costs. The development and application of this method will be discussed in conjunction with the derived benefits. In a process application where continuous monitoring is required, the operating cost of any technique is important. The relatively high costs associated with some TOC measurements can make on-line (process) TOC measurements very expensive. A method of measuring TOC inexpensively and rapidly would be a significant advantage in these applications. In this newly developed method, the oxidation is achieved through the use of titanium dioxide-based photocatalytic oxidation. Titanium dioxide is extremely inexpensive and also very safe. The oxidation takes place in a low-volume photocatalytic reactor that is specifically designed to provide highly efficient and rapid oxidation of organic carbon-to-carbon dioxide. Since the system does not require a carrier gas or other oxidizing reagents the operating costs are further reduced. The sensitivity of the system is vastly enhanced by the introduction of several miniaturized components, and an improved closed-loop flow design. The NDIR detector used in this method provides enhanced sensitivity while maintaining a small system volume. The final result is a unique TOC system that has a very low operating cost, hence making it particularly suited to continuous on-line measurements.

### Liquitoc-automated total organic carbon analyser with a uniquely designed combustion reactor

*Ralf Dunsbach[1], Joachim Kupka[1] and Scott Hughes[2], [1]Elementar Analysensysteme GmbH, Donaustrasse 7, Hanau D-63452, Germany, [2]Elementar Americas, Inc., 520 Fellowship Road, Ste B-204, Mount Laurel, NJ 08054-3408, USA*

Total Organic Carbon (TOC) analysers originated about 40 years ago: two techniques currently dominate, one using UV and the other high temperature for oxidation of the carbon in the sample. The current development uses the high temperature oxidation method for measuring TOC but is unusual because a multipurpose reactor compactly and efficiently houses several of the traditional chemical processes required in this analysis. The sample up to 2 ml flows into the vertical reactor. Acid is combined in the reactor converting the inorganic carbon (IC) to $CO_2$, which is then measured by the NDIR detector. Next a dynamic oven that surrounds the reactor heats the reactor from room temperature to 850 °C. in just < 2 min. Upon heating the non-purgeable organic carbon (NPOC) volatilizes and rises into the cerium dioxide catalyst housed in the upper chamber of the reactor heated by a second, static oven. The catalyst completes the oxidation so the $CO_2$ can be detected for the measurement of NPOC at the NDIR detector. Water samples with salt or other particles are by nature inhomogeneous thus demand high injection volumes. The reactor chamber can efficiently accommodate these high injection volumes. This approach offers a highly efficient system because the salt or other particles never come into contact with the catalyst avoiding typical problems of plugging and poisoning.

### Automated turbidity, conductivity, pH and alkalinity analysis of waters

*Aaron S. Beatty, Mandel Scientific, Product Development, 2 Admiral Place, Uelph, ON, Canada N1E 5Z1*

The task of ensuring that drinking water is safe has become increasingly important as fresh water supplies diminish and public awareness of the potential health hazards grow. Turbidity, the cloudiness of water, is an important general indicator of the quality of water. This cloudiness can come from suspended solids such as silt, clay, microorganisms, and organic and inorganic matter. Large amounts of these solids in drinking water reduce the ability of chemicals and radiation to neutralize disease-causing bacteria and viruses. The US Environmental Protection Agency (US EPA) highlights the importance of turbidity with regulations requiring that drinking water must have turbidity that is < 1 NTU, reported to the nearest 0.055 NTU, before being released for public use. The US EPA has also recently increased the number and frequency of turbidity samples required for municipal water treatment plants. Therefore, there is now a need for a precise and quick method for measuring turbidity. This need can be met with an automated turbidity analysis system. This paper will illustrate the performance of the automated Turbidity Assay-Plus™ meter, as an integrated module of the PC-Titration Plus™ Conductivity, pH and Alkalinity Analysis system. The parameters of time per sample, precision and detection limits will be discussed in detail.

### High-speed measurement of plant sterols by fast-GC to minimize sample degradation effects

*Gerald J. DeMenna[1] and James Kababick[2], [1]Chem-Chek Labs, PO Box 8307, 44 Stelton Road, Piscataway, NJ 08854-2600, USA, [2]Flora Research Labs, 31921 Camino Capistrano, 435 A, San Juan Capistrano, CA 92675-3210, USA*

Nutraceuticals has become the new 'golden bullet' in today's nature-oriented Pharmaceutical business. The AMA has finally begun to recognize nearly 4000 years of herbal medicines used by the ancient Chinese healers to treat everything from topical infections to mental disorders to menopause. Almost a dozen companies a month are opening their doors and producing products from the plethora of materials currently available WITHOUT restriction (soon to change with the upcoming QC requirements being proposed by the *United States Pharmacopoeia*); ranging from Echinaceia for treating colds to soy isoflavonoids as a hormone replacement therapy for menopause. Many of the active components for these complex formulations derive some of their activity from sterol-based compounds. A routine analytical methodology for both identification and quantitation of these materials is high-resolution Capillary Gas Chromatography using medium- to high-polarity column phases. Such runs typically take 30–60 min, and are prone to sample degradation due to the prolonged exposure to high temperatures during the separation. A technique using a column interface for direct heating of the capillary allows the same level of separation to be done 10 to 25 faster, and has the added advantage of maintaining the morphological integrity of the sample since the temperature exposure is so short. The EZ-Flash GC accessory is available for upgrading most existing GC systems, and was used for this investigation. Data from standard GC and EZ-Flash GC runs will be shown for comparison.

### Increasing laboratory efficiency with automated HPLC dissolution testing

*Patricia A. Fowler, Michael E. Swartz and Frasier H. Charles, Waters Corporation, 34 Maple Street, Milford, MA 01757-3696, USA*

The fast pace of the pharmaceutical industry requires laboratories to reduce the analytical burden of their test procedures, and increase productivity while still satisfying regulatory compliance. There are several ways to meet these challenges in the dissolution laboratory. By automating the dissolution process pharmaceutical laboratories eliminate the slight variations that may occur in manual methods, insuring reproducible data, higher throughput, and cost reduction. Validated single source software control of the entire system, as well as dissolution data acquisition, calculations, and reporting can further streamline the work flow while maintaining FDA compliance with 21 CFR Part 11. Automated on-line HPLC dissolution systems can pool dissolution samples significantly to save time. Similarly, automated sampling at shorter intervals and analysis of a large number of samples by on-line HPLC may provide a more complete solution for the decision making process in the early stages of drug development. In addition, these systems must be capable of handling increasingly complex formulations, such as multiple actives, and widely varying dosage levels, as well as different media types, such as buffers and surfactants. High-throughput applications including ultrafast chromatography and flow injection analyses can also significantly increase productivity. We will present new developments in an automated on-line HPLC issolution system that satisfies all of these challenges. Applications used to help reduce the analytical burden and increase the sample throughput and productivity in the laboratory will be highlighted.

### A virtual assistant for unattended development of HPLC methods

*Sergey Galushko[1], Oleg Pylypchenko[1], Vsevolod Tanchuk[2], Irina Shishkina[2] and Wolf-Dieter Beinert[3], [1]Institute of Bioorganic Chemistry and Petrochemistry of Nati, Fine Organic Synthese, Im Wiesengrund 49-b, Muehltal D-64367, Germany, [2]Institute of Bioorganic Chemistry and Petrochemistry of Nati, Murmanskaya 1, Kiev-94, Ukraine, [3]Merck KGaA, SLP Chromatography, Frankfurter Str 250, Darmstadt D-64271, Germany*

The aim of this communication is to demonstrate that an artificial intelligence program ChromSword Auto connected to an HPLC system can work as an autonomous virtual assistant developing HPLC methods. It plans experiments, runs an HPLC system, solves real method development problems and prepares reports. The program performs method development steps fully automatically, optimizing types and concentration of organic modifiers in a mobile phase, linear and multistep gradient profiles, temperature and even the choice of HPLC column. The program can solve automatically several separation problems. Typical examples are: development or optimization of final HPLC methods for chemical and pharmaceutical industry when all components of a mixture to be separated are available as standards. Development of HPLC isocratic and gradient methods when only some components of a mixture are known and available as standards. During measuring performance the system worked successfully in parallel with qualified chromatographers in pharmaceutical and chemical companies of Europe and USA carrying out real separation projects: (1) development of HPLC methods for quality control; (2) impurity and degradation products search and separation; and (3) related compounds separation. In many cases the unattended automated optimization led to better conditions than a trial-and-error procedure and requires much less human resources and user qualification then computer-assisted method development. The system is build in ChromSword-Auto software for automated method development (Merck KGaA, Darmstadt, Germany) and can control Merck-Hitachi and Waters HPLC systems.

### On-line redox derivatization for enhancement of separation selectivity of liquid chromatography

*Masami Shibukawa and Kazunori Saitoh, Nihon University, College of Industrial Technology, Department of Applied Molecular, 1-2-1 Izumi-Cho, Narashino 275-8575, Japan*

HPLC is one of the most powerful methods for separation of chemical compounds. However, the differences in retention factors of sample components on an HPLC column are often insufficient for complete separation. One of the ways to enhance the separation selectivity is the use of a chemical reaction specific for analyte compounds as a derivatization reaction. The derivatization for enhancement of separation selectivity is usually carried out batchwise before HPLC separation. On the other hand, we have recently developed a new HPLC system consisting of two separation columns and one redox derivatization unit placed between them. The redox reaction proceeds in the derivatization unit chemically or electrochemically so that an analyte compound is separated as its original form on the first column, while as its oxidized or reduced form on the second column. This system should enable one selectively to separate the analyte compound provided that only the analyte undergoes the redox reaction. We found out that a porous graphitic carbon was very useful as a catalyst for chemical redox derivatization. Its redox ability can easily be modified by treatment with a solution containing a suitable redox agent. We have also employed an electrolytic cell of which the working electrode is made of graphitic carbon as an electrochemical derivatization unit and have obtained successful results. This paper deals with development of the on-line redox derivatization HPLC method and its application to selective separation of some metal ions. The effects of applied potential flow rate and amount of analyte injected on the on-line derivatization efficiency of the system presented will be discussed.

### A novel FTIR imaging system combining an array detector with a rapid scanning spectrometer

*Richard Spragg[1], Robert Hoult[1], Andrew Turner[1], Ralph Carter[2] and Sarah Rahllf[2], [1]PerkinElmer Instruments, Post Office Lane, Beaconsfield HP9 1QA, UK, [2]PerkinElmer Instruments, Chalfont Road Seer Green, Beaconsfield HP9 2FX, UK*

Two approaches to generating IR images are currently being used. The combination of an MCT array detector with a step-scan spectrometer has allowed the production of high quality images in relatively short times, but at high cost. At the other extreme, mapping with a single detector, although much less expensive, has been prohibitively slow except for small images. New approaches are now beginning to emerge with the development of detector arrays designed specifically for this application. The availability of lower cost systems is expected greatly to expand the use of FTIR imaging. This paper describes the development of a simpler and less expensive FTIR imaging system using a rapid-scan spectrometer with a linear MCT detector array. The custom-designed detector combines in a single package the linear array, with a range to $720\,cm^{-1}$, and a single element detector covering the range to $600\,cm^{-1}$. Switching between imaging and conventional single point measurement does not involve moving any optical elements. The image is built up by moving the sample on a stepping microscope stage. In imaging mode, the pixel size can be either 6.25 or $25\,\mu m^2$. The system can image an area $2\,mm^2$ with $80 \times 80$ pixels in $< 2\,min$. This speed is comparable with that achieved with larger arrays, typically 500 times faster than point mapping. The image format can be matched to the application and the image size is limited only by the memory available. There are significant advantages to using a linear MCT array because it can operate in photovoltaic mode and have external electronics. This allows a wider spectral range and gives better signal-to-noise performance than larger arrays. The interferometer scan is linked to the movement of the stage so that data collection is uninterrupted. With simultaneous data collection from all the detector elements the duty cycle is considerably better than with larger arrays. One consequence of the excellent signal-tonoise performance is that images of specula reflectance can be recorded. Some issues that arise in mid-IR reflectance measurements will be discussed.

### The application of a new on-line trace boron analyser to control boron in a semiconductor ultrapure water system

*Richard D. Godec*[1] *and Rock Wickham*[2], [1]*Ionics Instruments, Inc., Sievers New Product Development, 6060 Spine Road, Boulder, CO 80301-3323, USA,* [2]*Integrated Device Technology, Facility Technology, 3131 NE Brookwood Pkwy, Hillsboro, OR 97124-5303, USA*

A new on-line trace boron analyser was used in at a semiconductor-manufacturing site to study the control of boron contamination in their ultrapure water. This presentation will review the new analyser technology, examine the data from the on-line boron analyser, analyse the effect of improved boron control, and compare laboratory ICP-MS boron measurements to the on-line analyser measurements of randomly collected samples. By using the new analyser, the manufacturer was able to show they could improve the control of boron contamination in the ultrapure water and save money at the same time.

### Using a roving robot with UV/visible-near-IR/IR imager for monitoring growth of cyanobacteria colonies

*Robert A. Lodder, University of Kentucky, Department of Chemistry, A123 Astecc Bldg, Lexington, KY 40506-0284, USA*

One branch of astrobiology is devoted to duplicating conditions of remote planets and moons on Earth, and identifying the life that survives there. Earth analogues for Mars are found in the deserts of Antarctica, and perhaps beneath glaciers. Cyanobacteria are one of the oldest forms of life on Earth, and similar forms of life may have developed on other planets like Mars as well. NASA has sent several missions to Mars to search for life, including the Mars Pathfinder mission. While the Pathfinder mission transmitted some of the best data obtained from the Martian surface to date, the long Martian winters and rough terrain limit the amount of data that can be obtained from different locations using a rover that must drive up to a target to sample it. The search for life near locations where water may be intermittently present demands ability to sense life farther from the rover. Hyperspectral imaging can be used to identify astrobiologically interesting sites from a distance. The rover can then drive up to the site and conduct further sampling. The cyanobacterium (blue-green algae) *Gloeocapsa* sp. produces a mycosporine-like compound that serves as a UV sunscreen in harsh environments. This compound can be used as a chemical marker for presence of colonies in the UV, visible, and near-IR spectral regions. Reflectance measurements made by a UV/visible-near-IR/IR imager mounted on an electrically powered robot were used to detect the growth of cyanobacterial colonies at distances up to 20 m. The image and spectrum below was obtained from a *Gloeocapsa* colony growing on a calcium carbonate substrate. (The spectrum of the limestone surface was subtracted.) The baseline distortion at the high wave number end of the spectrum arises from coloration of the colony that can be observed in the visible region. *Gloeocapsa* tolerate high-temperature and multiply in summer. Endolithic communities of *Gloeocapsa* can also be found in colder regions, including McMurdo Dry Valleys in Antarctica. While the absorptivity in the visible region is greater than in much of the near infrared, the near-infrared region yields a better 'fingerprint' for identification of spores.

### Prediction of chromatographic selectivity, retention times and peak widths for new capillary stationary phases and columns

*Frank L. Dorman*[1], *Chris English*[1] *and Paul Schettler*[2], [1]*Restek Corporation, Applications, 110 Benner CIR, Bellefonte, PA 16823-8497, USA,* [2]*Juniata College, Chemistry Department, 1700 Moore Street, Huntingdon, PA 16652-2196, USA*

For many years gas chromatographic (GC) stationary phases have been synthesized with no specific application in mind. Consequently, many analysts struggle to force a specific analysis to work on general-purpose chromatographic columns. The science of separation has been understood for some time, however, and capillary GC column scan be designed which achieve optimum separation for a known list of analytes. This paper will address the state-of-the-art design of capillary GCc stationary phases and describe how it can be used to improve radically the common separations that many chromatographers are faced with on a daily basis. Discussion will be given to the method of prediction of stationary phase selectivity, and how combining this with the calculation of peak width can drastically improve the phases that are used in modern separations. These techniques have the potential to allow the design of an optimum capillary column for any GC analysis.

### Chip-based DC glow discharge

*Andreas Manz, J. C. T. Eijkel, O. P. Naji, F. Bessoth, G. Jenkins and D. Reyes, Imperial College, Department of Chemistry, South Kensington, London SW7 2AY, UK*

Glass microstructures have been used for microfluidics, electrophoresis, flow-PCR, FIA and other applications during the last decade. More recently, plasma emission spectroscopy has been made available on chip, too [1–3]. The power requirements are in the 0.01–1.0 W range and atomic/molecular spectra can be obtained. Here we present the successful use of these chips for the analysis of Cl and Br containing VOCs, coupling them to GC, using a double plasma arrangement to create injected sample plugs [4], attempts to use them for water samples [5] and for analogue computing [6].

1. Eijkel, J. C. T. *et al. Analytical Chemistry*, 71 (1999), 2600.
2. Eijkel, J. C. T. *et al. Analytical Chemistry*, 72 (2000), 2547.
3. Engel, U. *et al. Analytical Chemistry*, 72 (2000), 193.
4. Manz, A. *et al.*, in J. M. Ramsey and A. van den Berg (eds), *Micro Total Analysis Systems* 2001 (Berlin: Kluwer, 2001), 655.
5. Manz, A. *et al.*, in J. M. Ramsey and A. van den Berg (eds), *Micro Total Analysis Systems* 2001 (Berlin: Kluwer, 2001), 349.
6. Manz, A. *et al.*, in J. M. Ramsey and A. van den Berg (eds), *Micro Total Analysis Systems* 2001 (Berlin: Kluwer, 2001), 37.

### Field-portable, fast GC/TOFMS

*Jack A. Syage, Brian J. Nies, Richard Harkewicz and Matthew D. Evans, Syagen Technology, Inc., 1411 Warner Avenue, Tustin, CA 92780-6461, USA*

This talk reports on a fast gas chromatograph, time-of-flight mass spectrometer (GC/TOFMS) for man-portable field use. The complete system is designed to meet performance, size, weight, power, cost and ruggedness requirements for a laboratory in the field. The core technology is also adaptable to specific applications including real-time point detection for hazardous chemical releases (e.g. chemical weapons), for biological agent signature identification, and for mobile monitoring platforms (e.g. air, ship, truck). In this talk we present recent progress in integrating a low-power, high-speed GC and show the capability for accurately recording fast GC transients for targeted compound detection using a photo-ionization (PI) source coupled to a quadrupole ion trap, time-of-flight (QitTof) instrument. The system specifications are 40 lb weight, 2 cu ft size, and 150 W of power consumption. The instrument records mass spectra at nominally 60 Hz (USA) making it suitable for fast GC. The GC is temperature programmable and can cycle typical samples in 60–240 s. Preliminary GC measurements gave baseline resolution with peak widths of about 1 s and detection limits of about 20–100 ppb and 10–50 pg.

### Miniaturization of analytical instruments: micro scale atomic spectroscopy

*Vahid Majidi, Los Alamos National Laboratory, CST-9, MS K484, Los Alamos, NM 87545-0001, USA*

The growing interest in process analytical and real-time chemical analysis for field applications has resulted in a new trend toward development of small analytical instrumentation. Improvements in materials possessing and electronics have resulted in a new breed of analytical instrumentation for off-the-shelf deployment. However, the strength of these instruments is mostly in the areas of spectrophotometry, electrochemistry and membrane-based sensors. These devices, in large, are used for one of two main applications: air monitoring or solution measurements. Over the last decade, the rapid development in the computer and electronic industries has lead to an overabundance of faster, cheaper and smaller microprocessors and ultimately fully functional computers. Considering the impact that computer control has had on laboratory-based analytical instrumentation, it is now possible to enjoy the same level of automation in field-based instrumentation. In addition to the vast improvements in the computational powers, the electronic circuits are now available in prepackaged forms to allow for modular instrument development. While plasma spectroscopy has a strong foothold in laboratory-based elemental analysis, it is nearly non-existent in field-based instrumentation. The paucity of filed deployable instruments for elemental analysis has been mostly due limitations in electronic, computational and optical technologies. Popular science magazines often depict future analytical instrumentation as microprocessor-sized devices, it is important to note that chemical analysis cannot be compacted to the same extent as their electronic counter parts. The size of an electron is infinitely small with respect to the submicron-diameter conductors in electronics. However, when considering miniaturization of chemical analysis systems submicron diameters of a capillary or sensor walls is not much larger than the size of the analyte of interest. For this presentation, two different micro plasma sources will be discussed. One system is based on atmospheric pressure plasmas while the second system is based on low-pressure plasmas. The goal of this work is not merely to miniaturize the plasma source but rather to design an integrated system, which takes advantage of scaling laws to reduce the size of every component.

### Detection of *Bacillus globigii* spores using a biochip system with miniature diode laser excitation

*Dimitra N. Stratis, Guy D. Griffin, David L. Stokes, Leonardo R. Allain, Joel Mobley and Tuan Vo-Dinh, Oak Ridge National Laboratory, Bethel Valley Road, Oak Ridge, TN 37830-8050, USA*

Owing to the increasing concern over the threat of biological warfare, there is a need to develop sensitive devices capable of early warning detection of biological warfare agents before human exposure. In particular, *Bacillus anthracis* (anthrax) spores are of great concern since they can be produced in large numbers, can be easily released into the air, and are highly resistant to inactivation. The work presented here will include the detection of *Bacillus globigii* spores (a surrogate for *B. anthracis*) using a prototype biochip system with photosensors, amplifiers, discriminators, and logic circuitry on board. To provide both a sensitive and selective analysis, an enzyme-linked immunosorbent assay (ELISA) for antibody-based capture of the target was used in conjunction with the biochip detection system. In this work, we use a novel alkaline phosphatase substrate, which produces a fluorescent product that is optimally excited at 635 nm upon enzymatic cleavage. Using an antibody-based assay with this substrate, we have demonstrated limits of detection of *B. globigii* of approximately 500 organisms. Similar results are achievable through enzyme amplification with DNA-based detection illustrating the ability to perform a sensitive analysis without conventional DNA amplification methods such as PCR. Since the product is conveniently excited at 635 nm, a small diode laser can be used. This along with the self-contained ORNL biochip design allows for a small, compact system for biological warfare detection.

### A biochip system with bubble-jet spotting technology for the detection of the fragile histidine triad (FHIT) gene in mice

*Leonardo R. Allain, Minoo Askari, David L. Stokes and Tuan Vo-Dinh, Oak Ridge National Laboratory, Bethel Valley Road, Oak Ridge, TN 37830-8050, USA*

The human fragile histidine triad (FHIT) gene has attracted considerable interest after its identification, since there is growing evidence that it acts as a tumour suppressor gene. In this work, the fabrication of microarrays containing PCR-amplified genomic DNA extracts from mice tumours on a Zetaprobe® membrane using a modified thermal inkjet printer will be described. The inkjet spotting constitutes a simple and cost-effective procedure for the fabrication of micro-arrays containing biological samples. The usefulness of the biochip micro-array platform is illustrated by the detection of FHIT gene. Subcutaneous carcinomas were induced with MKN/FHIT and MKN/E4 cell lines in immunodeficient mice. Several weeks into their development, the tumours from both groups of mice were removed, and subjected to DNA extraction by lysis of tissue samples. The extracted DNA samples were amplified by PCR (30 cycles) using appropriate primers for the FHIT sequence. The resulting solution was transferred to individual reservoirs of a three-colour cartridge from a conventional thermal inkjet printer (HP 694C), and arrays were printed onto a Zetaprobe membrane. After spotting, these membranes were used in a hybridization assay using fluorescent probes and detected with a CMOS biochip.

### Microfluidics: a modular approach to developing an analytical instrument

*Steve Hobbs, Gene Dantsker and Hugh McManus, Nanostream, Inc., 358 Sierra Madre Villa Boule, Pasadena, CA 91107, USA*

Microfluidic systems have the potential to revolutionize biochemical analysis and high-throughput screening. One major bottleneck in drug discovery is high-throughput separations. This talk will focus on the development of a microfluidic chip that can do multiple parallel separations with resolution approaching that of a standard HPLC. Data presented will demonstrate separations of proteins and small organic molecules. Detection with mass spectrometry and optical techniques will be discussed.

### Advanced automation interfaces: new toolkits for adding instrument control to a chromatography data system

*Dario Fiore and Kristi McKiney, Scientific Software, Inc., 6612 Owens Drive, Pleasanton, CA 94588-3334, USA*

One of the greatest limitations of chromatography data systems is in the area of true (interactive) instrument control for different types of instruments from disparate manufacturers. Because instrument manufacturers are in business to sell instruments, many feel it is not in their best interest to provide control for competitive instruments. Yet this means the user must learn to operate multiple data systems in a single lab, many of which might not be the data system of choice. Hundreds of man-hours are wasted in learning, maintaining, and validating different data systems that essentially do the same thing—data acquisition, analysis and instrument control. Many companies (both data system users and instrument manufacturers) are realizing the time and cost benefits of using open-architecture data systems where instruments of many brands are supported. Although adding control for an analytical instrument is not a task for the everyday user, new tools are now available that facilitate the interfacing of instruments to the EZChrom Elite Chromatography Data System, making it much easier and faster for instrument manufacturers and large companies to create an instrument control interface. The Toolkit automation interface provides the tools required for a qualified company to add instrument control, and the new Rapid Control product allows Active X control to be added to the Elite data system. This paper will describe these tools and give examples of their use.

### A collaborative environment for developers of scientific software

*Deborah Anne Kernan, Victoria Rafalovsky and Ty Abshear, Bio-Rad Laboratories, Informatics Division, 3316 Spring Garden Street, Philadelphia, PA* 19104-2552, USA

Bio-Rad's KnowItAll(tm) Analytical System offers an integrated environment for analytical techniques, such as IR, H-1 and C-13 NMR, UV/Vis, GC, MS, Raman, and NIR. Within this system, chemists can perform multiple tasks within the same interface, using software 'plug-ins' that reside within the main KnowItAll architecture. This design allows the user to easily transfer information from plug-in to plug-in without having to open another program. Because of this unique design, new third-party software plug-ins can be quickly and easily incorporated into the KnowItAll architecture. The philosophy of this unique architecture is one that built upon the ideal of getting solutions to scientists faster and giving them more options. Thus, through collaborations with database content providers and third-party software developers throughout the world, the system grows and adapts to meet the changing needs of the scientific community. For those parties with existing software that may fit within the content of the system, Bio-Rad has developed a simple Software Developer's Kit to convert software into a format that will 'plug into' and work within the KnowItAll system.

### Development and validation of analytical software in a regulated environment

*Kevin Bynum, Gillian Raymond, Lane Gehrlein and Philip Palermo, Purdue Pharma LP, Pharmaceutical Analysis, 444 Saw Mill River Road, Ardsley, NY 10502-2605, USA*

The development and validation of an analytical software package for use in the collection of cGMP compliant data will be discussed. The system has been designed to collect data to support drug development, stability testing, and release testing in the Pharmaceutical Industry. The software is designed to collect dissolution data from a UV probe fibreoptic dissolution system. The software, written in JAVA and C++, uses an Oracle database to ensure data integrity and security. The design features, which make this software 'validatable' will be discussed. The validation testing and implementation of the system on our corporate network will be discussed in detail.

### Validation Manager 2.0: new developments in computer-assisted assay methods validation

*Jean-Marc Roussel*[1], *Michel Righezza*[2] *and Wolf-Dieter Beinert*[3], [1]*Merck Eurolab S.A.S.*, 201 Rue Carnot, Fontenay Sous Bois F-94120, France, [2]*Antenne Scientifique Universitaire de Chartres, LBC, 21 Rue De Loigny La Bataille, Chartres F-28000, France*, [3]*Merck KGaA, SLP Chromatography, Frankfurter Str 250, Darmstadt D-64271, Germany*

For laboratories willing to meet the requirements of ISO 17025 regulations, analytical assay methods validation is an step important to consider. Recommendations for these method validations have been announced during the fourth International Conference on Harmonization (ICH 4) and the corresponding statistical calculation procedures are described in the ISO guidelines (i.e. ISO 5725, ISO 8466). Based on these recommendations, we have developed an assay methods validation software which allows validation planning, calculations and reporting according to the authorities requirements (USP, EP, FDA). Validation step consists in the study of the method characteristics as described in the guidelines and uses well-known and efficient statistical tests such as Dixon, Fisher, Student or Cochran. Although these statistical tools are frequently used in the analytical laboratories, most questions come when preparing the validation planning or determining the correct calculation configuration to be used. To help the analyst, we have implemented in the software ready to use validation templates which include, for different analytical techniques, predefined statistical tests confidence levels and default number of analytical results per validation characteristics, all these in conformity with the international recommendations and regulations. A validation document preparation wizard may be used in combination with these templates, in order to prepare the detailed validation planning in the easiest way. Electronic data security has become of the utmost importance, in this respect Validation Manager software proposes to the user a FDA compliant configuration in which every single addition or modification in data or result is fully documented and saved in a method logbook. Each versions of the validation document is saved in a specific folder and cannot be overwritten. Each data modification and calculation creates a new results version, ensuring full data integrity. Moreover, in order to avoid transcription mistakes, an automatic data recovery function has been developed allowing direct copy of information from the Hitachi D-7000 HSM and SSI EZ-Chrom Elite chromatography data systems. Using analytical data from our laboratory, the presentation will describe the different steps of method validation using Validation Manager. The next future of this development project, including the use of experimental designs for robustness study will also be presented.

### An automated software approach to analytical method validation

*Michael E. Swartz and Patricia A. Fowler, Waters Corporation, Pharmaceutical Marketing Lab, 34 Maple Street, Milford, MA 01757-3696, USA*

Method validation is a tedious process performed to determine if an analytical method meets the requirements for its intended purpose. In he regulated laboratory, method validation may take many days to perform the necessary analytical tests. Data reduction and the statistical analysis of results performed can be a very time consuming process. There is also a greater possibility of introducing error when calculations are performed manually. With the use of automated software to perform these calculations, method validation is much faster and easier, with less chance for error. In this presentation, we will show how an analytical method is validated using automated software. Chromatographic results are directly accessed from a relational database bypassing manual intervention. Statistical calculations are performed automatically and a report generated showing the results of the analyses from the Student, Cochran, Dixon, and Fisher Tests. Graphs are generated representing the results of the statistical analysis. In addition, we will show that the data reduction and statistical calculations necessary to validate the method, complete with the necessary documentation and report generation, are completed in significantly less time.

### An automated approach to developing HPLC methods for stability testing and related compound analysis

*Michael E. Swartz*[1], *Lloyd R. Snyder*[2], *John W. Dolan*[2], *Richard Lee-Berman*[2] *and Jupille Tom*[2], [1]*Waters Corporation, Department IM, 34 Maple Street, Milford, MA 01757-3696, USA*, [2]*LC Resources, Inc., 2930 Camino Diablo, Ste 110, Walnut Creek, CA 94596-3964, USA*

HPLC method development of complex pharmaceutical assays such as those used for stability testing or related compound analysis is a complex and time-consuming process. Trial and error is still a common approach, but many researchers prefer more efficient approaches that use chromatographic modelling software that relies upon theory to decrease the time and resources required. However, while automated HPLC systems exist to run the methods, there is a disconnection between the chromatographic and modelling software, resulting in a manual process requiring operator intervention for interpretation and implementation. We will present a new approach to HPLC method development automating the entire process from method requirements/definition to method implementation. The automated method development approach consists of both software and hardware operated by an iterative decision engine driven by a graphical user interface. Following input of basic separation requirements, starting conditions are identified and run, evaluated, optimized, and automatically implemented by the chromatographic system; and repeated until the separation goals are achieved. The result is an automated method development system capable of unattended operation increasing throughput and efficiency. We will demonstrate the use of this system for the development of complex pharmaceutical assays that take advantage of the selectivity afforded by high pH mobile phases and columns specifically designed for that purpose.

### Automatic HPLC method development with intelligent peak tracking

*Wolf-Dieter Beinert*[1], *Volker t Volker Eckert*[1], *Reinhold Spatz*[1], *Sergey Galushko*[2], *Irina Shishkina*[3] *and Vsevolod Tanchuck*[3], [1]*Merck KGaA, Scientific Laboratory Products, Frankfurterstrasse 250, Darmstadt D-64271, Germany,* [2]*Institute of Bioorganic Chemistry National Academy of Sciences, Fine Organic Synthese, Im Wiesengrund 49-b, Muehltal D-64367, Germany,* [3]*Institute of Bioorganic Chemistry of National Academy of Sciences, Murmanskaya 1, Kiev-94, Ukraine*

An automated expert system has been developed to search for optimum conditions in HPLC. Controlled by an artificial intelligence module, the system provides fully automated unattended method development in reversed-phase HPLC for mixtures of compounds and can search for the optimum concentration of an organic modifier for isocratic separation, for the best linear and multisegment gradient profiles and for the temperature optimum. For proper peak tracking, the first version of ChromSword Auto required single standards of the compounds to be separated. However, often one faces the situation that not for all compounds to be separated suitable standards are available. An example is the analysis of impurities in a product, where usually only a few (if any) of the impurities are available as pure substances. This is usually the case with pharmaceutical quality control samples for purity and stability tests. Therefore, ChromSword Auto has been equipped with intelligent peak tracking capabilities. Applying advanced mathematical procedures for peak assignment, it is now possible to perform the optimization procedure with the sample mixture. The number of standards necessary for the optimization procedure can be reduced to just two, regardless of how many compounds are present in the mixture. The objects of the automated optimization procedure are: (1) separation of the mixture into a maximum number of peaks (e.g. search for impurities) or separation of target compounds; (2) optimum resolution; and (3) minimum analysis time In this way it is possible to automatically optimize HPLC separations of chemical and pharmaceutical samples where only a few substances (e.g. the active compounds) are available as standards. ChromSword Auto thus makes possible dramatic timesavings in the method development process as well in routine analysis. Moreover, the resulting methods possess of this chromatography expert system will be explained by means of practical examples.

### The virtual assistant—a new HPLC system with expert knowledge and best performance

*Reinhold E. Spatz*[1], *Wolf-Dieter Beinert*[1], *Masahito Ito*[2], *Honori Kaji*[2] *and Richard Jack*[3], [1]*Merck KGaA, SLP Chromatography, Frankfurter Str 250, Darmstadt D-64271, Germany,* [2]*Hitachi Instruments Ltd, Biosystems, Hitachi-Naka 312-8504, Japan,* [3]*Hitachi Instruments, Inc., 3100 N. 1st Street, San Jose, CA 95134-1942, USA*

HPLC is by far the most used instrumental technique in the analytical laboratory. The instrument specifications continuously need to be adapted to new analytical requirements such as different column dimensions, high sample capacity, fast sample throughput, latest governmental regulations or state-of-the-art information management. The paper describes the concept of the newly developed modular LaChrom Elite HPLC instrument system. It is suitable as well for standard HPLC applications as well for special application segments in HPLC, like semimicro HPLC or high-throughput separations with monolithic stationary phases. The same system can be modified by the user without compromising specifications for the application field in target. This flexibility is due to a new hardware concept and due to integrated unique software options that act like a ‘virtual assistant’ in the background to deliver highest expertise whenever necessary for a certain application. Typical examples that require optimized hardware are semimicro HPLC with 1 mm i.d. columns or high throughput HPLC with monolithic columns and flow gradients in the 10 ml $min^{-1}$ flow rate range. Typical examples for Virtual Assistance are integrated software modules such as ‘ChromSword Auto’ for fully unattended HPLC method development or ‘AutoValidation’ for automated Operation and Performance Qualification (OQ, PQ) of the HPLC system for regulated Quality Control Laboratories. The paper will describe the new hardware and software concept. Many evaluation results and typical applications will prove the high technical level of this new development.

### Identification of protein folding subunits by ion mobility-mass spectrometry

*Brandon T. Ruotolo, Kent J. Gillig, Earle G. Stone and David H. Russell, Texas A&M University, Department of Chemistry, PO Box 30012, College Station, TX 77842, USA*

There have been several theories proposed as to the mechanism of folding processes in proteins. Among them, the idea that certain peptides segments of a protein exhibit intrinsic stability and contain the site(s) of helix nucleation within the protein, i.e. autonomous folding subunits, has been proposed and observed in a few isolated cases. However, with the introduction of relatively new methods for probing the conformation of biological molecules in the gas phase, such as ion mobility spectrometry, additional information can be obtained on the validity of this hypothesis. Proof-of-concept experiments have been performed on tryptically digested proteins, which are then screened by MALDI-IM-TOF MS. For example, a peptide signal from a tryptic digest of horse heart myoglobin was observed to deviate by more than 10% in total drift time from the other peptides in the IM-MS map. The peptide was sequenced using tandem mass spectrometry and identified as the majority of the E helix of solution phase myoglobin. This peptide was found to exhibit helical structure in simulated annealing molecular dynamics simulations. This presentation will focus on our recent efforts to probe the ability of MALDI-IM-TOF MS to screen for the presence of these extraordinary peptides. The updated screening protocol involves digestion with a number of different proteolytic agents and in different solvent systems in order to produce a more complete map of the protein under investigation. These results will be discussed in light of insight gained on protein folding mechanisms.

### Quantitative in-line measurements of paper coatings by near infrared

*Jon G. Goode[1], Qian Wang[1], Angela Schmidt[2] and Helmut Weiler[2], [1]Bruker Optics, Applications, 19 Fortune Drive, Billerica, MA 01821-3923, USA, [2]Bruker Optik, Near Infrared Applications, Rudolf-Plank-Strasse 23, Ettlingen D-76725, Germany*

Leading manufacturers in the paper industry are looking for in-line methods to determine the physical properties, perform component analysis and determine applied coating weights in paper. The MATRIX-E, an in-line process control FT-NIR spectrometer, makes possible a non-contact diffuse reflectance measurement with a large sampling area. We will discuss an on-line paper application to determine the silicone coat weights on labelstocks. This is currently performed in the laboratory by a time-consuming X-ray fluorescence method. The aim was to achieve an absolute value for the deviation from the target value of 1 g m$^{-2}$ during continuous paper production at velocities of approximately 400 m min$^{-1}$. Concentrations of silicone between 0 and 2 g m$^{-2}$ on various paper substrates were included in a quantitative model and influences from the uncoated paper type due to supplier, colour, opacity, area densities, precoatings as well as different compounds of the silicone agent were investigated. It was found that all these factors could be included in a single PLS-model. The fact that elemental silicone is present in clay-coated papers was found to be of no consequence to the measurements with MATRIX-E. Moreover, during in-line installations it was found that the variation of the moisture content in the moving paper due to variable machine velocities as well as the reflecting material of the cylinder had to be considered. It will be shown that the result of the in-line calibration has the same prediction capability as lab scale results (root mean square error of cross-validation RMSECV = 0.034 g m$^{-2}$).

### Process monitoring of the moisture and finish-on-fibre of textile products using a portable near infrared analyser

*James E. Rodgers[1], Rafael L. Barraza[1], Panitan Sukpaladisai[2] and Charles M. Horton[3], [1]Solutia, Inc., 3000 Old Chemstrand Road, Cantonment, FL 32533-8926, USA, [2]Solutia, Inc., Highway 20 West, Decatur, AL 35609, USA, [3]Solutia, Inc., 1515 Highway 246 S., Greenwood, SC 29646-8402, USA*

The moisture content and the quantity of finish applied to the surface of textile fibres (Finish-on-Fibre, or FOF) are often critical process and quality control variables, for they can significantly impact physical properties, manufacturing processes, quality, and productivity. A recurring problem for textile bobbin products is the rapid detection and identification of outlier moisture and FOF bobbins during production. Non-contact, at-line moisture and FOF measurements directly on the bobbin in manufacturing would result in improved yields, process monitoring, and Quality Assurance. In this work we establish the feasibility of using a portable Near InfraRed (NIR) moisture analyser (Kett KJT100) to measure moisture directly on nylon tire and carpet yarn bobbins in manufacturing and demonstrate the feasibility of a subjugate process monitoring measurement of bobbin FOF during spinning. NIR moisture calibrations were developed for numerous tire and carpet products (different yarn type, size, finish, etc.) at three locations (laboratory, lag area, spinning). The NIR method successfully monitored moisture differences between bobbins in the laboratory and manufacturing areas. In spinning, a strong correlation was observed between NIR bobbin moisture and FOF. Subjugate FOF calibrations for tire and carpet products were developed in spinning, and excellent agreement was observed between the NIR and reference FOF results. of critical importance to manufacturing was the rapid at-line identification of several 'outlier' bobbins, preventing their contamination of downstream processes. The impacts of yarn parameters, measurement location, and environmental and operational conditions on the NIR results were slight.

### Novel applications of Raman spectroscopy to process monitoring and materials' characterization

*Brian J. Marquardt, CPAC, Center for Process Analytical Ch, FJ-20, Seattle, WA 98195, USA*

This presentation will focus on the use of Raman spectroscopy for the analysis of solid samples (powders, slurries, etc.) with emphasis on on-line process analysis applications. A novel high precision Raman probe for on-line process analysis will be described. The unique design of the Raman probe provides enhancements in measurement precision by increasing the reproducibility and accuracy of optical sampling of high solid content samples. The probe has been proven an effective sampling interface for the analysis of powders, suspensions, slurries, particles and solids. The ease of use of the Raman probe and the increased sampling precision has lead to its use in various proofs of concept and on-line process analytical applications. These applications include dry powder–powder mixing efficiency, coating thickness measurements, and solvent drying analysis, reaction monitoring and various other analytical processes. In this presentation I will discuss the physical and optical design of the Raman probe and demonstrate its applicability as an on-line sampling tool.

### Determination of sulfhydryl residues in cysteine-rich metalloproteins using flow-injection quartz crystal microbalance

*Alejandro Lopez Briseno, Alfred Joe Baca, Fayi Song and Feimeng Zhou, California State University, Los Angeles, Chemistry and Biochemistry, 5151 State University Drive, Los Angeles, CA 90032-4226, USA*

Metallothioneins (MTs) are low-molecular weight proteins having amino acid compositions rich in sulphur which strongly bind to metal ions such as Zn, Cd, Cu and Hg. Extensive research has been conducted concerning the two types of redox groups (metals and mercaptide groups) in these molecules. Particular emphasis has been aimed at determining the number of sulfhydryl groups involved in the electron transfer reactions of metallothioneins. However, less attention has been directed in the determination of sulfhydryl groups that are not directly involved in the redox properties of these proteins. We report here our recent efforts in determining the number of sulfhydryl groups that directly participate in the redox reactions of Metallothioneins. It was found that approximately four cysteines per MT molecule are involved in the cysteine–mercury thiolate formation. This was accomplished by combining two well-known techniques: the electrochemical quartz crystal microbalance (EQCM) and the inductively coupled plasma atomic emission spectrometry (ICP-AES). Another objective of this study will be to determine the availability of non-participating cysteine groups by signal amplification through chemical reaction to convert sulfhydryl groups into easily detectable moieties. A conceivable method of accomplishing this is by selective modification onto cysteine moieties acting as probes to methoxy-polyethyleneglycol maleimide (MPEG-MAL). The quartz crystal microbalance (QCM) will be used for gravimetric analysis of the sulfhydryl groups.

### Miniaturized flow-through surface plasmon resonance detector for the study of protein–protein interaction dynamics

*Rebecca J. Whelan[1], Thorsten Wohland[1], Richard N. Zare[1], Lars Neumann[2], Jacqueline Steenhuis[2] and Brian Kobilka[2], [1]Department of Chemistry, Mudd Chemistry Building, Stanford, CA 94305, USA, [2]Departments of Medicine Cardiol, Beckman Center, Stanford, CA 94305, USA*

Surface plasmon resonance (SPR), which senses changes in the refractive index of a dielectric medium adjacent to a thin gold film, is a powerful tool for label-free studies of biological molecules and their interactions in real-time. We employ SPR to study the interaction of the G-protein-coupled beta-2 adrenergic receptor with other molecules, including receptor agonists and antagonists, kinases, and G. protein. The most relevant information comes from receptors that are immobilized on the surface in a uniform way. To this end, we have developed a novel mutation of the receptor, with an additional cysteine at the extracellular terminus. Biotinylation of this mutant receptor and use of conventional biotin/avidin binding fixes the receptor to the gold film of the SPR sensor with excellent uniformity. An alternate immobilization protocol uses an antibody (M1) directed against the receptor's amino-terminal FLAG tag. The receptors retain their function after immobilization, as confirmed by fluorescence microscopy studies. After introduction of a reaction partner through a flow cell (operating with or without continual flow), information can be obtained about the extent of interaction, binding kinetics, and pharmacology. Other biological interactions we have investigated include antigen/antibody, glycoprotein/lectin, and ssDNA/ssDNA. Regardless of the analyte system, maximum sensitivity and efficiency are achieved by minimizing the volume of sample above the sensing surface. To minimize the required sample, we have developed a miniaturized flow cell. This small flow cell also opens the possibility of coupling SPR with a miniaturized separations platform such as CE or micro-HPLC.

### Screening for environmental contamination using liquid chromatography with mass spectrometry detection

*Jim Krol and Kate Yu, Waters Corporation, 34 Maple Street, Milford, MA 01757-3696, USA*

When assessing unknown contamination of an environmental sample, where does the chemist begin? Which validated method is appropriate, a specific analyte method or several different analyte methods? What if the validated method reports no contamination; productivity decreases, analysis cost increase, and the contamination analyte remains unknown. Liquid chromatography (LC) has the capability to retain and separate numerous analytes based upon their chemical properties, but conventional PDA-UV detection has limitations in seeing all the analytes. For analyte identification, retention time alone is not sufficient. Chromatographic analyte coelutions will exist in complex samples and compromise UV spectra making identification and quantitation marginal. If the analyte is UV inactive, analyte identification and quantitation are impossible. Mass spectrometry (MS) is a more universal, yet specific detection technique that detects a significantly greater number of environmental analytes, such as carbamate and urea-based pesticides and herbicides using positive electrospray. Combined with gradient reverse phase chromatography, an environmental sample can be screened for numerous analytes. The simultaneous use of retention time, $m/z$ mass spectra, and PDA-UV are used as qualitative variables for analyte identification. However, the low ppb semiquantitative results need to be confirmed by specific validated methods. This presentation will present a novel carbamate and urea analyte screening method approach. The critical link is the reliability of the MS processing method, chromatographic reproducibility, and the percentage of false-positives/false-negatives. Wastewater and ground water will be the evaluated matrices.

### Fast gas chromatography with conventional instruments using direct resistively heated capillary columns

*Paolo Magni, Giacinto Zilioli and Riccardo Facchetti, ThermoQuest Italia SpA, Research and Development, Strada Rivoltana, Rodano, Milan I-20090, Italy*

In gas chromatography, there is an increasing demand for significant reductions in analysis time. However performing high-speed separations while keeping enough separation efficiency is a difficult task. The combined use of fast temperature programming and short narrow bore columns may provide an optimal solution, particularly for samples containing simple mixtures with a wide boiling point range. For some applications a sufficient separation can be achieved even in < 1 min provided that, in the same time, the column is raised to an appropriate final temperature. The system presented in this paper, consisting in a directly heated capillary column module mounted in a conventional GC instrument, permits to achieve very fast temperature programming rates (as high as 20 °C $s^{-1}$), which cannot be obtained with the use of conventional air circulating

GC ovens. The tremendous heating rate power of the device permits to reduce the tight bandwidth sample injection requirements otherwise dictated by the small internal diameter of the column. The module, containing the capillary column, the heating element, and the temperature sensor, is housed in the GC oven and connected to standard GC split-splitless injector and detection systems, just as any conventional capillary column. Results obtained with both split and splitless injection techniques are examined using Flame Ionization Detector and Time of Flight Mass Spectrometer. The attached chromatogram shows an example of fames analysis, ranging from n-C7 to n-C22 fames, performed in $< 1$ min. The separation was obtained using a 5 m, 0.1 mm i.d., 0.1 $\mu$m film thickness RTX-5 column with a temperature-programming rate of 5 °C $s^{-1}$ (from 70 to 300 °C). Others applications of the fast GC accessory in the chemical, petrochemical, environmental, and food and flavours fields are presented.

### Modification of OSHA GC methods for continuous area monitoring

*John N. Driscoll, Process Analyzers, LLC, 25 Walpole Park South Drive, Walpole, MA 02081, USA*

OSHA has developed hundreds of GC methods for analysing organic compounds in the workplace as part of the standards process. These methods were developed to provide a analytical methods for samples collected on adsorbent tubes in the workplace for a wide variety of industries. Typical sample volumes collected range from 250 ml (at the ceiling value) to 12 litres (below the TLV) of sample collected on the adsorbent tubes. Once the sample (250 ml) is diluted, it is equivalent to injecting a 1 $cm^3$ sample at approximately 5 the TLV. For area monitoring, we would like to have a detection limit that is 1/20th of the TLV or PEL. If these detectors were used in an Automatic GC for Area monitoring, many would have difficulty detecting half the TLV for those compounds with low TLVs. In other words, many of these methods would be 'sensitivity challenged' because of the use of the flame ionization detector (FID). These OSHA GC methods can still be used but it clear that a more sensitive method (or a concentrator for the FID) is needed in order to obtain the required sensitivity. The photo-ionization detector (PID) is greater than 50 times more sensitive than an FID for aromatic compounds. This will allow a smaller sample (0.1–3 $cm^3$) to be used and still have adequate sensitivity for the method. In addition, the selectivity of PID can be varied by choosing a 9.5, 10.2 or 11.7 eV lamp. The sensitivity of the FID can be increased via an on-board automatic concentrator that can improve the detector sensitivity by 10–100-fold. The compounds to be concentrated can be optimized by switching the trap materials. Another difficulty that occurs at low ppm or ppb levels with polar species is adsorption or reactivity on/with surfaces of the sampling system and lines. This results in serious problems with reproducibility and accuracy. Examples include amines, phenols, organic and inorganic acids, pesticides. A permeation tube or other diffusion device can be used to condition the entire system and prevent adsorption of polar or reactive compounds. We will evaluate a number of methods for amines, sulphur compounds, chlorinated hydrocarbons and diphenyl oxide. We will compare these results of the FID/NPD detectors with the photo-ionization detector (PID) mounted on an automatic GC for area monitoring. This should provide an alternate method that has more sensitivity and/or selectivity and does not require support gases.

### Modification of OSHA GC methods for continuous area monitoring

*John N. Driscoll, Process Analyzers, LLC, 25 Walpole Park South Drive, Walpole, MA 02081, USA*

OSHA has developed hundreds of GC methods for analysing organic compounds in the workplace as part of the standards process. These methods were developed to provide analytical methods for samples collected on adsorbent tubes in the workplace for a wide variety of industries. Typical sample volumes collected range from 250 ml (at the ceiling value) to 12 litres (below the TLV) of sample collected on the adsorbent tubes. Once the sample (250 ml) is diluted, it is equivalent to injecting a 1 $cm^3$ sample at approximately 5 the TLV. For area monitoring, we would like to have a detection limit that is 1/20th of the TLV or PEL. If these detectors were used in an automatic GC for area monitoring, many would have difficulty detecting half the TLV for those compounds with low TLVs. In other words, many of these methods would be 'sensitivity challenged' because of the use of the flame ionization detector (FID). These OSHA GC methods can still be used but it is clear that a more sensitive method (or a concentrator for the FID) is needed to obtain the required sensitivity. The photo-ionization detector (PID) is $> 50$ times more sensitive than an FID for aromatic compounds. This will allow a smaller sample (0.1–3 $cm^3$) to be used and still have adequate sensitivity for the method. In addition, the selectivity of PID can be varied by choosing a 9.5, 10.2 or 11.7 eV lamp. The sensitivity of the FID can be increased via an on-board automatic concentrator that can improve the detector sensitivity by 10–100-fold. The compounds to be concentrated can be optimized by switching the trap materials. Another difficulty that occurs at low ppm or ppb levels with polar species is adsorption for reactivity on/with surfaces of the sampling system and lines. This results in serious problems with reproducibility and accuracy. Examples include amines, phenols, and organic and inorganic acids, pesticides a permeation tube or other diffusion device can be used to condition the entire system and prevent adsorption of polar or reactive compounds. We will evaluate a number of methods for amines, sulphur compounds, chlorinated hydrocarbons and diphenyl oxide. We will compare these results of the FID/NPD detectors with the photo-ionization detector (PID) mounted on an automatic GC for area monitoring. This should provide an alternate method that has more sensitivity and/or selectivity and does not require support gases.

### An automated system for combined sample preparation and analysis for high-throughput screening

*Kerstin Thurow, Agnes Schubert and Christian Wendler, University Rostock, Institute of Automation, R-Wagner-Str 31, Rostock, MV, D-18119, Germany*

The use of combinatorial methods in chemistry and life science has been developing rapidly within the last years. One of the 'bottlenecks' in synthesis control in pharmacy and life sciences is still the characterization and speciation of biotechnological reaction products using chromatographic methods in combination with specific detection systems. Often before analysis a complex sample preparation is required which might include cleaning steps, dilution or even derivatization procedures. Existing systems are not flexible automated solutions and do not have flexible material and information interfaces. Thus, the objective of our investigation was the development of an automated system for combined sample preparation and analysis being used for organic synthesis. The system used is a commercial liquid handling station (CTC CombiPal), which has been equipped with different trays for heating, filtration, mixing, etc. and has been adapted to different analytical devices such as gas or liquid chromatographic systems or mass spectrometers. Technically, the system is controlled by a digital computer operating both chromatograph and sampler as different tasks in the WINDOWS-NT 4.0 operating system. The sample preparation task allows the application of user-specific methods. Sampler and chromatograph communicate for transmitting remote START/STOP-information via hardware handshake lines. Because of the flexible system strategy, the system can be set up easily for different applications. The system developed allows the fully automatic analysis of samples to run in parallel with the sample preparation. It can be used as a stand-alone system or can be integrated in complex robotic systems. Motivation for such a combination is to reduce further testing costs by increasing throughput and by reducing manual intervention. Other advantages include, for example, reducing the delay between the analysis and the sample preparation. The presentation will outline the technical details of the system developed and will show the use of the system for high-throughput screening applications in the field of combinatorial catalysis, chiral determinations and synthesis control.

### Development of a high-throughput analysis infrastructure for combinatorial materials science applications

*Radislav A. Potyrailo, General Electric, Corporate Research and Development, PO Box 8, Schenectady, NY 12301-0008, USA*

High-throughput methods for materials science combine a parallel or combinatorial materials synthesis and processing with an automated screening and data-management tools. They can rapidly optimize molecular properties and process conditions that are difficult to predict using existing knowledge. From an analytical chemistry perspective this leads to major challenges in developing rapid analysis techniques that can deal with large numbers of small samples required to construct combinatorial libraries. To meet these analytical challenges in the discovery of new materials at General Electric, a high-throughput analysis infrastructure was established at GE's Corporate Research Center. This presentation will describe the strategy taken in the development of this infrastructure. For high-throughput analysis of materials properties from a wide variety of projects, analytical instrumentation consists of interchangeable modular subsystems coupled with the new data processing capabilities. Examples from several projects will illustrate the broad applicability of the developed high-throughput analysis instrumentation.

### High-throughput analytics employing automated sample preparation and the integrated use of HPLC-DAD, HPLC-DAD-MS, GC-MS and flow injection NMR

*Winfried Etzel[1], Stefan Beeck[1] and Wolfgang Gau[2], [1]Bayer AG, Agrochemical Division, Chemical Research GEB 6530, Leverkusen D-51368, Germany, [2]Bayer AG, Landwirtschaftszentrum Monheim, Alfred-Nobel-Str 50, 6530, Monheim D-40789, Germany*

The establishment of automated and parallelized synthesis and microscale reactions in the chemical synthesis laboratories of the Agrochemicals Division at Bayer created an increasing demand for analytical information (purity, structure, physicochemical properties). With the availability of flow injection NMR systems (BEST-NMR) the use of the same sample format on different analytical instruments was possible. This fact encouraged us to realize an integration of different analytical techniques (NMR, HPLC-DAD, HPLC-DAD-MS, GC-MS) in one service unit. The optimization of the flow NMR system for the use of different organic solvents will be demonstrated and the quality of the spectra and the throughput of samples will be discussed. In addition, the results and advantages of an integration of different analytical techniques will be presented. Workings with barcodes and microtitreplate (MTP) technology were introduced. Electronic laboratory journal, sample preparation robotic system and analytical instruments were connected together via a laboratory information and management system (LIMS). This resulted in a simplified workflow in the chemical research laboratories and in the analytical department. The throughput of samples and measurements was doubled without raising the number of technicians.

### Ultrafast liquid chromatography: the importance of system temperature

*Jon D. Thompson and Peter W. Carr, University of Minnesota, Department of Chemistry, 207 Pleasant Street SE, Minneapolis, MN 55455-0431, USA*

It is very important that the speed of HPLC be increased. Improving throughput for stability analysis, quality control assays, and dissolution testing are all key motivations for improvement in separation speed in the pharmaceutical industry. Unlike CE, HPLC does not lend itself well to multiparallel analysis because the cost is prohibitive. Recent work in this lab has focused on the theory of high temperature on analysis time in LC. Disregarding selectivity considerations, we have shown that the five to 10-fold decrease in effluent viscosity that comes from working at very high temperature (180–200 °C), and the concomitant increase in analyte diffusivity, combine to dramatically decrease the time needed to generate a theoretical plate. The lower viscosity at elevated temperature decreases the pressure drop across the column and allows higher linear velocities to be reached as the pressure limit of the pump is approached. Simultaneously, faster analyte diffusion at higher temperature improves efficiency at high linear velocity conditions compared to the efficiency at lower temperatures at the same velocity. In this presentation, we will show how the theory of speed guides column selection and system design. We will show that at high temperatures, 10 gradient runs can be done in 40 min on columns of conventional geometry. We will also show that dramatic improvements in stability indicating assay throughput can be achieved at elevated temperature using conventional equipment.

### A new stand-alone autosampler for high-throughput HPLC and HPLC/MS

*Curtis R. Campbell, Susan M. Steinike and Kara M. Merkle, Shimadzu Scientific Instruments, LCMS Applications, 7102 Riverwood Drive, Columbia, MD 21046-1245, USA*

Fast LC and fast LCMS methods have been developed to meet the demands of combinatorial chemistry and drug discovery. Now more than ever, is there a corresponding requirement for ultrafast sample introduction in LC and LCMS instruments along with the most stringent requirements for zero carryover and optimum sample repeatability. In this communication we describe the performance of a newly designed, stand-alone autosampler for HPLC and HPLC/MS applications featuring a 15-s injection cycle for 10-μl injections, excellent sample repeatability, and the lowest carryover currently attainable. The unit can function independently, allowing interface with a variety of instrument models in a variety of venues including combinatorial chemistry, drug discovery, and high-throughput screening.

### Control of low-frequency noise in inductively coupled plasma optical emission spectroscopy by on-line periodic reference standard correction

*Michelle E. Cree[1], James Barker[2], Ross Ashdown[2] and Dimitry Profus[2], [1]Varian, Inc., 25 Hanover Road, Florham Park, NJ 07932-1410, USA, [2]Varian Australia, 679 Springvale Road, Mulgrave, Victoria 3170, Australia*

The understanding of ICP–OES instrumentation has attained a level where various engineering, chemical and mathematical techniques have been implemented to improve the precision of analytical results. The remaining sources of noise in an ICP-OES spectrophotometer can be broken into high- and low-frequency noise. High-frequency noise in a simultaneous ICP–OES can be corrected by the use of 'real-time' internal standardization. The ability to control the effects of high-frequency noise has seen the effects of low-frequency noise now dominate the precision of results. Drift from low-frequency noise can be defined as a slow varying change in the background or intensity of a signal while the instrument is performing analysis. Until now, the primary method used to produce 'drift free' results was to compensate for an instruments slow, varying change during analysis by performing constant recalibrations. An alternative method is to perform data processing externally, using third party software. Both methods are time consuming, and decrease productivity. Now a faster more economical approach uses 'real-time' drift correction. Varian has implemented a real-time drift correction technique that allows long-term collection of data, with little loss in precision due to drift. Just as internal standardization was first implemented and precision was improved, now drift correction will further improve the precision of analytical results. To obtain the most precise results, two drift models are available, linear and polynomial. Both use defined drift correction points to complete mathematically a goodness of fit correlation, with the polynomial using progressively higher orders of fit until the threshold acceptance criteria is established. Here we will look at the performance of both models.

### Quasi-continuous monitoring of process and waste streams by on-line inductively coupled plasma–optical emission spectroscopy

*Dirk Ardelt, Heinz Falk, Hendrik M. Smol and Hans-Joerg Waarlo, Spectro Analytical Instruments GmbH & Co. KG, Boschstrasse 10, Kleve D-47533, Germany*

Over the past decade, increasingly more sophisticated process analysers derived from 'mature' laboratory methods of instrumental analysis have found widespread use in the chemical process industry to cope with increasing demands for more efficient energy and material usage and better process control. Although many such applications still rely on rather simple sensors, an increasing amount of formerly laboratory based only methods, like Infrared—or Raman-Spectroscopy, X-ray—Fluorescence or magnetic resonance methods, are nowadays routinely applied as on-line systems. As diverse as these methods are their fields of application in the on-line process environment, which typically is divided among the distinction between the aggregate states of the medium to be analysed. Not surprisingly, one major field is the analysis of liquid process- and waste streams. Considering laboratory use, inductively coupled optical emission spectroscopy, ICP-OES, probably does the most universal atomic spectroscopy method exist. Combining high flexibility, good sensitivity, wide dynamic range and fast measurement cycles, this method should ideally be suited for migrating into the process analytical field, especially regarding its relative 'maturity'. Despite of these findings, ICP-OES up to now has not found the expected use as on-line analyser, even where it outperforms other methods under laboratory conditions. Using recently installed systems for wastewater and process stream monitoring as examples, we will discuss probable reasons for the rather low past development of on-line ICP-OES and examine, in how far these reasons have been or can be overcome. Relevant analytical and technical figures of merit for on-line applications shall be deduced. Furthermore, modern multichannel detection ICP-OES allows for using sophisticated algorithms (e.g. chemometrics) for data evaluation, in this case resulting in higher degrees of automation and system availability. Accounting for such advances, we will try defining a 'state of the art' for ICP-OES as process analytical method and highlight future development areas.

### Millisecond monitoring of fast transient signals by Hadamard transform time-of-flight mass spectrometry

*Joel R. Kimmel*[1], *Facundo M. Fernandez*[2], *Jose M. Vadillo*[2] *and Richard N. Zare*[2], [1]*Department of Chemistry, Mudd Bldg, 333 Campus Drive, Stanford, CA 94305-4401, USA*, [2]*Department of Chemistry, Mudd Chemistry Building, Stanford, CA 94305-5080, USA*

Hadamard transform time-of-flight mass spectrometry (HT-TOFMS) involves the modulation of a continuous ion beam using a pseudo-random sequence of pulses [1]. This sequence of pulses is applied using a charged particle modulator consisting of an interleaved comb of wires. Ions flying from the electrospray ion source to the flight chamber are 'gated' at this modulator following the Hadamard-type binary sequence. The applied sequence has an approximately equal number of zeros and ones, thus the duty cycle of the spectrometer is close to 50%. TOFMS has become the preferred detector for separation techniques used in bioanalytical chemistry such as CE and LC. As research continues, the obtained separation efficiency of these methods increases, making the eluting peaks sharper. To describe correctly the shape of these narrow peaks, detectors must acquire data at a rapid speed. The only means of increasing the spectral acquisition speed of a TOFMS without sacrificing mass range is to use encoding methods that eliminate the lag time between successive ion packets. In this presentation, we describe the ability of HT-TOFMS to scan transient signals in the millisecond time-scale. A brief theoretical background on how TOFMS can be multiplexed is given. Alternative Hadamard transform algorithms that improve the performance of slow multichannel scalers are discussed in terms of their effect on the spectral storage rate. The effect of acquisition systems' end-of-pass dead time is examined. The ability of HT-TOFMS to scan transient peaks of decreasing width is investigated for CE and pulsed electrospray ionization peaks. For femtomole injections of biomolecules, full spectra in a mass range of 1500 Da are obtained in 3.6 ms. Owing to the extremely large amounts of data collected, only state-of-the-art acquisition systems can be used for this task. Further increase in the spectral storage rate can only be achieved by further increasing the duty cycle of the instrument. The possibility of using two MCP detectors to produce a 100% duty cycle with no ion storage is discussed.

1. Zare *et al. Journal of the American Society of Mass Spectrometry*, 12 (2001), 1302.

### Automated sample spotting on Maldi plates

*Alan Hamstra, Tim Hegeman and Luke Roenneburg, Gilson, Inc., 3000 W. Beltline Hwy, Middleton, WI 53562-1617, USA*

Sample preparation is a rate-limiting step in the analysis of samples with MALDI-TOF instrumentation. Plate preparation requires, adding both sample and matrix, allowing them to form a homogenous mix and dry. Knowing the limitations of hardware, sample concentration, sample volume and sample density helps predict sample thought put. Hardware is available with positional accuracy to < 100 μm, however positional accuracy is only one parameter affecting the sample area occupied on a plate. MALDI plates come in a variety of sizes and configurations, placing requirements on sample application hardware. The volume of available sample imposes additional limits on hardware. The calculated theoretical diameter occupied by a 100 nl sphere is 726 μm. When placed on the plate surface the drop compresses vertically and occupies a diameter > 726 μm. Plate surface chemistries, either hydrophobic or hydrophilic, also affect plate sample density. Matrix and sample surface tension and viscosity affect plate area occupied by sample. To pass through the small probe orifice matrix and sample must be free of any particulate or a probe will plug and dispense inaccurately. Required sample accuracy and carryover specifications place time limitations on plate preparation. Loading a 384-well plate with matrix requires only minutes, while rinsing probes between samples to prevent carry over requires from 20 to 60 min. A rinse cycle may take significantly longer than sample application and account for 50–90% of the time required to load a plate. Technology is available to dispense nanolitre volumes of sample. However, there are limits on the sample volume, which can be aspirated. If minimal aspirated sample volume is limited to microlitres, each dispense of nanolitres may waste microlitres. Hardware and software must recognize this limitation and take steps to conserve sample. I will present data on minimizing the area occupied by sample, optimizing sample volume and mass, and minimizing plate preparation time.

### Software and services in Internet timescales

*Mark E. Fish, Thermo LabSystems, 1 St George's Court, Altrincham WA14 5TP, UK*

With the advent of the Internet one of the main characteristics of the modern business environment is the accelerated speed at which things can happen. A term 'Internet time' has been introduced to describe this phenomenon. 'Internet time' is personified by the boom, bust and rise again of the dot.com industries, where changes that would have previously taken years can occur in the period of a few weeks. A key reason for this is that Internet has dramatically advanced the ability of businesses to access and publish information. Informatics systems have focussed on data capture and information management, but the effect of Internet time is refocusing business efforts on providing efficient and cost-effective access to information and knowledge locked in enterprise systems. This time-shrinking effect means it is increasingly difficult for traditional product and implementation lifecycles to provide close fit solutions in a world of such rapidly changing businesses and requirements. Delivering laboratory centric solutions in 'Internet time' creates new challenges for professional services organizations and requires new approaches. This paper will examine these challenges and the place of both software and services in providing Internet solutions in Internet timescales.

### Sample management for DNA testing laboratories

*Nick Townsend, Martin Waugh and Peter B. Mansfield, Autoscribe Ltd, 81 Technology Park Drvie, East Falmouth, MA 02536, USA*

DNA technology has revolutionized applications such as paternity testing, forensics, clinical diagnosis and the detection of Genetically Modified Organisms (GMOs). This success has in-turn created a very large increase in the number of samples being processed by DNA testing laboratories and the associated logistical issues this can bring. In the last two decades LIMS have become established supporting the work of analytical and research laboratories but is it only recently that DNA testing laboratories been able to realize the benefits LIMS can deliver. In this presentation we will describe a LIMS configured to meet the needs of DNA contract testing laboratories. We will describe how, via web technology, clients submit samples for testing, specify the DNA testing profile and confirm order details. Once samples are received, bar code technology is used to track sample location and process steps including extraction, purification, amplification and identification. A very graphic and intuitive interface is provided for technicians allowing creation and editing of plate and electrophoresis gel plans using drag and drop techniques. The system supports the use of complex 'plating rules' providing a large degree of automation as to how primers, samples and reference standards should be pipetted onto the plates. A comprehensive audit trail is provided recording details of how samples are analysed including the run conditions associated with processing the plates and the transfer of samples from plates to gels. Once analysis is complete the LIMS automates the production of reports for the client and will interface with order processing systems to deliver an invoice for the work.

### Multivariate analysis in chromatography: peak tables versus profiles

*L. Scott Ramos, Infometrix, Inc., PO Box 1528, Woodinville, WA 98072-1528, USA*

Chemometric software tools can detect the presence of meaningful information in chromatographic data and create multivariate models able to interpret future chromatograms. Commonly, tables of integration results are processed using nearest neighbour or factor-based classification algorithms. Complex applications benefit from the increased prediction accuracy and sensitivity to outliers (e.g. unusual samples, process upsets). However, peak tables have limitations: peak assignments are prone to error when peaks elute closely, peaks that show only as shoulders are not integrated, and peaks not in the calibration table are ignored. Now that computational power is cheap and ubiquitous, processing of whole chromatographic profiles is attractive. Using whole profiles eliminates the disadvantages cited for peak tables. Nevertheless, profiles have distinct requirements for reliable use: chromatograms must be aligned so that peaks of the same material are found at exactly the same time, the data collection rate must be considered—100 scans across a peak is unnecessary over sampling, and, finally, very large numbers of lengthy profiles in a database can overwhelm even the most capable computers. This paper will contrast the two approaches, highlighting the advantages and disadvantages of each. Examples from different applications will demonstrate the relative merits of the two approaches.

### Detection of heroin in drugs of abuse using multivariate curve resolution with two-dimensional wavelet compression

*Guoxiang Chen[1] and Peter de B. Harrington[2], [1]Ohio University, Department of Chemistry & Biochemistry, Clippinger Laboratories, Athens, OH 45710-2979, USA, [2]Ohio University, PO Box 1625, Idaho Falls, ID 83415-0001, USA*

A trend in contraband drug manufacturing has been to adulterate popular drugs such as cocaine and MDMA (ecstasy) with heroin. The adulterants are added to either increase the potential of addicting the user or as an introduction to harder drug use. Ion mobility spectrometry (IMS) is used for screening drug samples by law enforcement agencies. A chemometric procedure was developed for rapidly detecting heroin in other drug samples. Multivariate curve resolution techniques have been developed mathematically to separate the components in mixtures. The self-modelling curve resolution method SIMPLe-to-use Interactive Self-modeling Mixture Analysis (SIMPLISMA) decomposes a data set into a model that comprises concentration profiles and spectra. SIMPLISMA has proven an effective tool for simplifying intricate ion chemistry that may occur in IMS data. In this work, a SIMPLISMA algorithm with two-dimensional (2D) wavelet compression was developed to analyse rapidly the data sets of drugs, including heroin, cocaine and MDMA (3,4-methylene-dioxy-methamphetamine). The 2D wavelet compression, which compresses both acquisition time and drift time dimensions of a measurement, was applied to the drug data sets before SIMPLISMA modelling. In comparison with the 1D compression, the multidimensional compression furnished a greater compression of data set size without losing important chemical information. This feature allows faster implementation of SIMPLISMA, improves the SIMPLISMA models by removing noise from the data, and lowers storage capacity that makes real-time modelling possible. The SIMPLISMA model obtained from the compressed data was wavelet transformed to the uncompressed representation. The effects of wavelet filter types and compression levels were investigated. The relative root-mean-square errors of SIMPLIMSA spectra, which calculate the relative difference between the extracted models with and without 2D compressions, were used to evaluate the effects of compression on self-modelling. The results showed that satisfactory models could be obtained at a compression ratio as high as 99.6%.

### Enzymatic assays on microchip platforms

*Joseph Wang, New Mexico State University, Department of Chemistry and Biochemistry, PO Box 30001, Las Cruces, NM 88003-8001, USA*

Microfabricated fluidic devices, integrating the sample preparation process and the measurement step onto microchip platforms, are of considerable recent interest. Such technology offers great promise for developing versatile and miniaturized analytical microsystems with high degree of automation, rapid analysis times and negligible consumption of reagents. Separation microchips have been combined with chemical derivatization reactions, but rarely to enzymatic processes. In this presentation, we will report on the versatility of microfluidic devices for carrying out novel multi-enzyme assay protocols and for tailoring the fluidics to suit specific bioassays. These will include the use of mixed oxidase/dehydrogenase preseparation reactions, or post-capillary derivatizations using multiple oxidases or dehydrogenases. Such on-chip enzymatic assays have been combined with fast electrophoretic separation and amperometric detection of the liberated NADH and hydrogen peroxide. We are currently exploring a multichannel operation, involving different combinations of enzymes, for high-throughput measurements of numerous substrates.

### Comparison of multivariate calibration techniques for NIR and FTIR data sets

*Larry McDermott[1], Bruce McIntosh[1], Martha Ranc[1], David Jantz[1] and Lauren Stewart[2], [1]Orbital Sciences Corporation, Applied Instrument Technologies, 2771 N. Garey Avenue, Pomona, CA 91767-1809, USA, [2]Orbital Sciences Corporation, Advanced Monitoring Development, 1 Bethel Valley Road, Oak Ridge, TN 37830-8050, USA*

A data set of over 500 North American gasoline samples was used to investigate various algorithms to determine if improvements in accuracy can be obtained for real-time measurements using on-line analysers. Algorithms investigated include multiple linear regression, locally weighted regression, neural networks, K nearest neighbour, principal components regression (PCR), partial least squares (PLS1 and PLS2) and non-linear regression techniques. Results obtained for key parameters including the research octane number (RON), motor octane number (MON), distillation points, aromatics, olefins, benzene and others will be discussed. Commercially available chemometric modelling software as well as proprietary developed algorithms using Matlab will be used for this study.

### Study of tellurium and selenium in environmental samples by pneumatic nebulization and hydride generation inductively-coupled plasma mass spectrometry; sensitive and interference-free determination of a high ionization potential element (Se) by hydride generation inductively coupled plasma mass spectrometry operated in a warm plasma condition

*Karl X. Yang, Kamal Swami and Liaquat Husain, Wadsworth Center/NY State Health Department, New York Statehealth Department, Corning Tower Empire State, Room D315, Albany, NY 12201, USA*

An inductively coupled plasma mass spectrometer operated in low rf power (700 W) was demonstrated to be suitable for ultratrace determination of elements with high ionization potential. Hydride generation was used for sample introduction. Selenium analysis free from $ArAr^+$ interference was achieved by operating the plasma at a so-called critical power condition where a warm (as opposed to traditionally defined hot and cool plasma) plasma was believed to exist. In this warm plasma, there is sufficient population of energetic $Ar^+$ to ensure ionization of Se while the $ArAr^+$ (interfering with the Se isotope) population is at the minimum due to its bimodal profile. Detection limit of 1 ng $l^{-1}$ was achieved for $^{78}Se$ without substantial interference from $^{78}ArAr^+$ after optimizing the operating parameters. Background spectra of warm and hot plasma were compared. Two types of hydride generation gas–liquid separators were compared. This technique was applied to measure trace amounts of Se in water extracted aerosol samples with typical concentrations between 50 and 100 ng $l^{-1}$. Recoveries of the independent 50 ng $l^{-1}$ QC and the SRM1640 (Natural Water) were 98 and 101%, respectively. It is conceivable that this technique can be used on other high ionization elements (e.g. As and Sb) if the critical power condition can be found with these elements.

### High productivity of environmental sample analysis using ICP-MS

*Jenny Godfrey and Phil Shaw, Thermo Elemental, Ion Path, Road Three, Winsford CW7 3BX, UK*

Elemental analysis of environmental samples in survey and monitoring programmes, must meet various criteria. These include cost-effectiveness, robustness, high-throughput, and fit-for-purpose accuracy and precision. The characteristics of environmental samples collected in these programmes are dominated by the variability of the sample matrix. Samples with widely differing major element composition and total dissolved solids content are necessarily analysed within the same batch. This makes the possibility of using matrix-matched standards very difficult. The technique of choice needs to be multi-element, matrix tolerant, have wide dynamic range, rapid data acquisition and good washout characteristics with appropriate detection limits. To be cost-effective, an instrument needs to be analysing samples for $> 12$ h a day whilst retaining the desired characteristics. This requires reliable hardware and versatile software needing minimal user intervention. An ideal system would be able to run environmental samples with little daily maintenance, e.g. cone cleaning, and allow direct transfer of data into a LIMS without further processing. The current generation of ICP-MS instrumentation and software is capable of performing drift corrections, monitoring QCs and recalibrating accordingly, monitoring washout of sample and applying corrections for internal standards, dilution, blanks and interferences on-line. This paper will describe new strategies to obtain high productivity and describe methodologies for running high matrix environmental samples to achieve world-wide environmental protocols by ICP-MS. With data shown on improved detection limits and recoveries, handling of interferences and maintaining high-throughput of multi-element samples.

### Total and individual assays of nitrated explosives on a simple microchip platform

*Martin Pumera*[1] *and Joseph Wang*[2], [1]*New Mexico State University, Chemistry & Biochemistry, Horseshoe MSC 3C, Las Cruces, NM 88003, USA,* [2]*New Mexico State University, Department of Chemistry and Biochemistry, PO Box 30001, Las Cruces, NM 88003-8001, USA*

Because of the upsurge in terrorist activity, there are urgent needs for fast-responding ultrasensitive portable devices for on-site detection of major explosives. A chip-based analytical system is presented that allows rapid flow-injection measurements of total nitro-aromatics explosives, as well as micellar chromatographic identification of the individual ones in the same microchannel. Fast-responding field deployable explosive analysers, providing a timely warning and alarm (in case of sudden concentration change), are being developed in our laboratory. A unique and rapid 'switching' between the 'non-separation' (ultrafast screening, analysis time.

1. Wang, J., Pumera, M., Chatrathi, M. P., Escarpa, A., Musameh, M., Collins, G., Mulchandani, A. and Lin, Y. *Analytical Chemistry* (2002) (in press).

### Monitoring cellular release using electrochemical and fluorescence detection on a microfabricated electrophoresis chip

*Drew P. Manica*[1,2], *Julie A. Lapos*[1] *and Andrew G. Ewing*[1], [1]*Pennsylvania State University, Chemistry, 152 Davey Laboratory, 49, University Park, PA 16802, USA,* [2]*Pennsylvania State University, Department of Chemistry, 2010 Malott Hall, Lawrence, KS 66045-7564, USA*

As microchip analytical systems continue to advance, their use as separations-based sensors for monitoring biological processes is increasing. One biological process that has received much attention for its role in neurological disorders is exocytosis, the process of neuronal communication. A method for monitoring exocytosis with microchip electrophoresis and electrochemical detection is demonstrated. Classical methods of monitoring cellular release involve massive dilution, or whole cell lysates. Microchip analysis systems provide the advantages of high speed and throughput, portability and low cost, while providing a convenient way to load low-volume samples, including cellular contents, directly onto the device. Release of neurotransmitters from small numbers of invertebrate neurons is performed in a total volume of a few microlitres, thereby reducing the effects of dilution. Then the extracellular fluid is injected, and the electro-active neurotransmitters are detected at a microfabricated platinum electrode. Initial experiments suggest the feasibility of the system to monitor vesicular neurotransmitters. Future directions will include employing dual electrochemical and fluorescence detection on a microchip in order to broaden the selectivity of detectable neurotransmitters and to aid in monitoring the extracellular environment.

### Multichannel separations with optically gated fluorescence injection on a chip

*Hongwei Xu*[1,2], *Andrew G. Ewing*[1], [1]*Pennsylvania State University, Chemistry, 152 Davey Laboratory, Davey L2, University Park, PA 16801, USA,* [2]*Pennsylvania State University, 1134-2 Gongendo Satte-Shi, Saitama 340-0193 Japan*

With the advantages of low analytical sample volumes needed and short separation times, microfabricated capillary electrophoresis chips have provided high-efficiency separations of DNA, proteins and other biological molecules. Also, the ability of fabricating multiple lanes on a single chip make it possible to parallel process many samples on one chip and reduce the total analysis time. The multiplexing of multiple channels on one microchip is complicated by the need for multiple injections. We have developed a technique that uses optical gating injection with multiple channels leading to high-throughput separations. Current work in our group has shown the ability of optical gating for sample introduction on a single channel chip. In optical gating, one laser beam is used to photobleach the fluorescent-labelled sample and injection is carried out by blocking this beam. The other beam detects the fluorescence from the injected sample after separation. Optical gating provides fast, serial injections and easy automation. With this technique, massive parallel separations in multiple channels can be carried out on a single chip. Combing optical gating and multiplexed channels will allow high speed, high-throughput separations for biological analysis, including genetic polymorphisms, proteins and others. In this technique, the chip is fixed on a stage and a voice coil actuator is used to move the stage back and forth so that the two stationary beams (gating beam and detection beam) can scan all the channels on the chip. Initial separations of amino acids and oligonucleotides have been used to determine the promise of the technique.

### Investigation of fluorescence polarization as a novel detection for improved DNA sequencing on a microchip

*Yuan Yan*[1,2], *Hong Li*[1] *and Michael L. Myrick*[1], [1]*University of South Carolina, Department of Chemistry and Biochemist, 631 Sumter Street, Columbia, SC 29208-0001, USA,* [2]*University of South Carolina, Department of Chemistry—C-2, 1, 207 Pleasant Street SE, Minneapolis, MN 55455-0431, USA*

Current four-colour DNA sequencing with fluorescence dye-labelled primers or terminators is the method most commonly used. Labels are chosen on the ability of a fluorimeter or suitable band-pass filters to distinguish their colours. Current technology for the one-lane four-colour approach limits the number of dyes that can be effectively distinguished under sequencing conditions. Recently, we have demonstrated that fluorescence polarization could be used to discriminate the isochromatic dyes in fluid solution [1, 2]. Fluorescence polarization will be further studied as a novel detection for increased selectivity of dyes while simultaneously maintaining sensitivity in this report. DNA sequencing is performed on a microfluidic device under a home-made confocal fluorescence research system. Some characteristics such as speed, accuracy and feasibility of this approach will be reported as well.

1. Yan, Y. and Myrick, M. L. *Analytical Chemistry*, 73 (2001), 4508.
2. Yan, Y. and Myrick, M. L. *Analitica et Chimica Acta*, 441 (2001), 87.

### DNA analysis on a microfabricated poly(dimethylsiloxane) chip with integrated electrochemical detection

*Nicole E. Hebert and Werner G. Kuhr, Department of Chemistry, Pierce Hall, Riverside, CA 92521-0001, USA*

The goal of this research is to develop a microfluidic chip using capillary electrophoresis and sinusoidal voltammetry for the separation and detection of small biomolecules. Biomolecules frequently require derivitization when detected by fluorescence or electrochemistry, the two most common detection schemes used on-chip. Derivitization is time consuming and can produce interfering compounds, which further complicate the analysis. However, it has been shown that under alkaline conditions, biomolecules such as carbohydrates, peptides, and DNA undergo electrocatalytic oxidation in their native state at the surface of copper electrodes, at potentials ranging from 0.4 to 0.8 V versus Ag/AgCl. This leads to high sensitivity for the detection of DNA [1]. Copper electrodes are fabricated onto a glass substrate, which are sealed reversibly to a poly (dimethylsiloxane) (PDMS) slab which contains micron-sized channels. A problem in the fabrication and assembly of such chip-based devices is leakage from the separation channel along the electrode surface, which can lead to increased background and band broadening. To optimize the electrode geometry and reduce background, a variety of polymers have been used to define the electrode area. Other parameters, such as thickness of deposited electrodes, techniques of assembling and filling small diameter channels, and composition of PDMS have also been explored. To evaluate the performance of the microfluidic chip, a simple, well-established system is used. The analysis of carbohydrates in free solution is a model system for the optimization of the above mentioned parameters. Upon characterization, the separation and detection of native oligonucleotides will be demonstrated.

1. Singhal, P. and Kuhr, W. G. *Analytical Chemistry*, 69 (1997), 4828.

### DNA sequencing using time-resolved fluorescence detection in a PMMA microchip

*Li Zhu and Steven A. Soper, Louisiana State University, Department of Chemistry, 232 Choppin Hall, Baton Rouge, LA 70803-0001, USA*

Time-resolved fluorescence detection has been proved to be a viable technique for the applications in DNA sequencing. To achieve high-speed separations and miniaturization of the devices, PMMA-based microchips are used in this study. In the first part of this research, a one-colour microscope system with a single channel PMMA chip (effective separation distance of 10 cm) is used. Cy5.5 and IRD700 dye-labelled primer and sequencing ladders will be separated and the lifetimes will be obtained using a 3%T/3%C polyacrylamide sieving medium, which is polymerized within the microchip channel (the replaceable linear polyacrylamide gel has been studied by another member in our group). Time-correlated single photon counting device is used for lifetime measurements. The other part of this research is using a dual colour microscope system (680/780 nm excitation), in which fluorescence lifetimes of two different dyes (IRD700/IRD800) will be simultaneously obtained. This microscope will be first tested on PMMA single channel chips and then mounted on a translational stage to read fluorescence from a 16-channel microchip which is constructed in our laboratory. In addition, techniques for filling microfabricated channels with denaturing polyacrylamide matrices, loading DNA sequencing samples on a chip, injecting samples, and the optimization of separation conditions will be discussed. Results of this research will significantly prove the feasibility of performing high-speed genomic sequencing using microfabricated electrophoretic devices and advantages of using lifetime detection.

### Monitoring biomolecule interactions at a single molecule level

*Gang Yao[1,2], Xiaohong Fang[3] And Weihong Tan[4], [1]Department of Chemistry, Gainesville, FL 32603, USA, [2]Institute of Chemistry, 52 Sanlihe Road, Beijing, 100080, P. R. China, [3]Department of Chemistry & The McKnight, PO Box 117200, Leigh Hal, Gainesville, FL 32611-7200, USA, [4]PO Box 549, Livonia, LA 70755-0549, USA*

We have imaged the process of biomolecule interaction and reactions at the single molecule level. Immobilizing single molecular beacon DNA molecules onto a solid surface and monitoring their fluorescence signal during the hybridization to their complementary DNA achieve this. Fluorescence images are taken using a total internal reflection fluorescence microscopy equipped with an intensified charged couple device (ICCD). It is found that many individual molecular beacon DNA probes have a gradual fluorescence increase within about 20 s. This indicates the conformational change of the hairpin shaped molecular beacon from stem close state to open state happens synchronously with the process of the loop hybridization. Our result also shows that the intermediates with partially base pairing between the two single strands may exist during the hybridization de-spite the well known all-or-none mode of DNA duplex formation. We found that the hybridization increases the photostability of the dye in a double-stranded DNA. We have also used the same technique for DNA/protein interaction studies on a single molecule level.

### Analytical instrumentation and LIMS: a fully integrated approach

*Charlotte Culley, Thermo LabSystems, 1 St George's Court, Hanover B, Altrincham WA14 5TP, UK*

The majority of LIMS today have some kind of interface for directly reading results from instrument data systems, hence avoiding manual transcription errors, etc. However, there are problems associated with losing the link to the original analytical data files. This paper will discuss progress towards an integrated system where the LIMS reads results directly from the instrument system, while at the same time gathering the corresponding data files etc and storing them in the same secure LIMS environment. This approach has the two benefits. First, from an Electronic Record Keeping perspective it ensures that the actual data used to generate the results in the first place has true referential integrity with the results. Secondly, from a Knowledge Management perspective, other information relating to the instrument data is available to the LIMS user, allowing big increases in productivity when it comes to sharing and mining information.

### Making our LIMS your LIMS—quickly!

*Peter B. Mansfield and Clive Seaton, Zumatrix, Inc., 81 Technology Park Drive, East Falmouth, MA 02536-4442, USA*

When a laboratory embarks on a LIMS implementation the process typically begins by referring to a user requirement document that was prepared many months earlier which may not always reflect current needs. As the vendor and customer begin detailed implementation work, both parties usually reach a new level of understanding of what the LIMS is required to do. As the implementation progresses, the LIMS is moulded to meet the user requirements—a process which often requires a measure of compromise by both parties depending on the flexibility of the LIMS. Once the LIMS is in production it will be expected to have a lifetime extending over many years and, during this time there is likely to be numerous requests to modify or extend the capabilities of the system. In this presentation we will describe an elegant suite of LIMS configuration tools designed to facilitate the smooth implementation of a LIMS to meet the current user requirements. We will describe how this suite of tools can also be used to modify the LIMS as business and/or laboratory needs evolve. A 'wizard'-style interface and use of a LIMS configuration knowledge base helps the vendor, and later the system administrator, create and/or modify end-user screens. Other tools facilitate documenting the configuration and providing configuration control. Successful LIMS do not stand still and the presentation will explain how a new generation of LIMS configuration tools will deliver significant business benefits throughout the lifetime of the system.

### DNA profiling and LIMS integration

*Phil Goddard, CSols, Inc., 13 Bolton Road East, Port Sunlight CH62 4RU, UK*

The integration of instruments with LIMS is well established for traditional analytical techniques such as chromatography, spectroscopy, etc. Standard, commercial software products exist (such as Links for LIMS produced by CSols) which can handle this type of link in a straightforward manner. Such software clearly handles the mechanistic functions of reading and writing data from/to the instruments. However, it also carries out many specific, technique-dependent tasks, which are necessary to automate laboratory processes and make it usable in real world situations. There has been a recent and rapid growth in biotechnology techniques and instrumentation. These instruments provide few additional problems for linking software with respect to reading and writing data. However, their technique-dependent requirements are very different from those demanded by traditional analytical instruments. This lecture examines what instrument integration software has to handle in one specific biotechnology example DNA profiling to make it a usable tool for transferring useful data to LIMS. The application is an important one as it is widely used in forensics, paternity testing and wide range of R&D environments. Specific automation issues are discussed including: acquiring peak data from the instruments, identifying true allelic peaks, recognizing and dealing with spurious peaks and known experimental artefacts (e.g. Stutter peaks, N-Peaks, Pull-up Peaks), checking standards, presenting and managing data in an effective manner, communication with LIMS. The example is used to highlight the types of additional issues that are important when linking biotech instruments to LIMS. There is also a discussion on what extra features it has been necessary to add to Links for LIMS to produce a Biotech Version.

